# Flexural wave-based soft attractor walls for trapping microparticles and cells†

**DOI:** 10.1039/d0lc00865f

**Published:** 2021-02-09

**Authors:** Amirreza Aghakhani, Hakan Cetin, Pelin Erkoc, Guney Isik Tombak, Metin Sitti

**Affiliations:** a Physical Intelligence Department, Max Planck Institute for Intelligent Systems, 70569 Stuttgart, Germany; b Electrical and Electronics Engineering Department, Özyeğin University, 34794 Istanbul, Turkey; c Faculty of Engineering and Natural Sciences, Bahcesehir University, 34353 Istanbul, Turkey; d Electrical and Electronics Engineering Department, Boğaziçi University, 34342 Istanbul, Turkey; e Institute for Biomedical Engineering, ETH Zurich, 8092 Zurich, Switzerland; f School of Medicine and School of Engineering, Koç University, 34450 Istanbul, Turkey

## Abstract

Acoustic manipulation of microparticles and cells, called acoustophoresis, inside microfluidic systems has significant potential in biomedical applications. In particular, using acoustic radiation force to push microscopic objects toward the wall surfaces has an important role in enhancing immunoassays, particle sensors, and recently microrobotics. In this paper, we report a flexural-wave based acoustofluidic system for trapping micron-sized particles and cells at the soft wall boundaries. By exciting a standard microscope glass slide (1 mm thick) at its resonance frequencies <200 kHz, we show the wall-trapping action in sub-millimeter-size rectangular and circular cross-sectional channels. For such low-frequency excitation, the acoustic wavelength can range from 10–150 times the microchannel width, enabling a wide design space for choosing the channel width and position on the substrate. Using the system-level acousto-structural simulations, we confirm the acoustophoretic motion of particles near the walls, which is governed by the competing acoustic radiation and streaming forces. Finally, we investigate the performance of the wall-trapping acoustofluidic setup in attracting the motile cells, such as *Chlamydomonas reinhardtii* microalgae, toward the soft boundaries. Furthermore, the rotation of microalgae at the sidewalls and trap-escape events under pulsed ultrasound are demonstrated. The flexural-wave driven acoustofluidic system described here provides a biocompatible, versatile, and label-free approach to attract particles and cells toward the soft walls.

## Introduction

Acoustofluidic devices have attracted growing interest in biophysical, biochemical and biomedical applications.^1–7^ In these acoustically driven devices, the method of manipulation of microparticles and cells is called acoustophoresis, where the motion of particles and cells are carried out by the acoustic radiation and the streaming-induced drag forces. In many studies, the acoustophoretic manipulation of microparticles and organisms, such as separation,^8,9^ focusing,^10,11^ sorting,^12,13^ patterning^14,15^ and trapping of particles,^4,16–18^ using different designs have been presented.

In such applications, surface acoustic wave (SAW) and bulk acoustic wave (BAW) based microfluidic channels are the most commonly used techniques. In SAW-based designs, piezoelectric solid substrates (usually made of LiNbO_3_) have been used as the acoustic waveguide source where upon actuation the sound waves propagate along the substrate, termed as travelling SAWs, and transfer the acoustic energy into the fluid medium. The numerical models and experimental works have been analyzed in detail for the SAW-based microfluidic devices.^19–25^ In general, the width of the channels is designed by one to ten times of the wavelength of SAWs, where the drive frequencies lie in the range of 10–1000 MHz.^26–28^ In BAW-based systems, however, a piezoelectric transducer is attached to the bottom of a solid substrate in order to excite the transverse modes, where the microfluidic channel is sandwiched between the carrier and the reflector. The standing wave is then generated by the superposition of incident and reflected waves travelling on the substrate where a large part of the wave is reflected inside the fluid channel. This causes the acoustophoretic motion of particles/cells inside the channel. Numerous experiments and numerical analyses have been presented for the BAW-based microfluidic devices, where the width of the channels is designed by a half of the wavelength of BAWs with the drive frequencies in the range of 1–10 MHz.^29–35^

For commonly used BAW-based devices, the ultrasonic standing wave in a microfluidic channel occurs between the opposing silicon walls, which act both as the fluidic and acoustic boundary. In such devices, there is maximum pressure on the walls due to the presence of hard silicon walls, which makes the wall trapping limited.^17^ Though, in sub-wavelength resonator configurations, a carefully designed resonator-reflector setup allows positioning of nodal pressure point at the reflector side. This way, particles/cell can be pushed towards one side of the wall, which is usually the reflector.^16,36,37^ Leibacher *et al*.^17^ proposed a modified BAW-based system with additional polydimethylsiloxane (PDMS) layer between the hard walls and the fluid channel. By choosing the thickness of the PDMS layer, they were able to adjust the pressure nodes at the boundary for collecting the particles. Nevertheless, this complicates the fabrication process and requires an accurate design of the impedance-matched soft PDMS layer. For standing SAW devices, the pressure nodes lie inside the microchannel due to the small acoustic wavelength,^38^ and the acoustic radiation force directed at Rayleigh angle pushes the particles toward the microchannel ceiling. This effect was utilized by Collins *et al*.^39^ to locally trap the microparticles at a thin membrane integrated into the ceiling of a microchannel *via* vertical force component of travelling SAW. Most recently, a diffraction-acoustic SAW-based manipulation method was proposed, where the particles with positive acoustic contrast factor were pushed to the channel edges.^40,41^ The acoustic manipulation of particles or cells by pushing them toward the wall surfaces has a significant role in enhancing immunoassays and particle sensors as thoroughly reviewed by Wiklund *et al*.,^4^ and recently in the field of microrobotics.^42^ In this regard, previous BAW-based and SAW-based devices utilize acoustic wavelengths comparable to or smaller than the channel size. As an alternative, flexural waves with wavelength much larger than the channel size could provide an effective wall trapping of particles and cells at the sidewalls of a microchannel. Earlier works on exploiting flexural modes for particle manipulation include the vibrating glass plate design of Haake and Dual.^43,44^ In their study, the resonance modes of the glass plate were used to concentrate particles in one or two dimensions. Similarly for SAW devices, lamb waves of piezoelectric substrate were used for particle manipulation within liquid drops.^45^ Recently, Ahmed *et al*.^42^ have demonstrated the migration of superparamagnetic aggregates to the PDMS microchannel walls using the acoustic radiation force and rolling along the boundaries by a rotating magnetic field. Their system comprised of a PDMS microchannel on a glass slide with an adjacently bonded piezoelectric transducer to generate the acoustic waves. Although the similar setup has been often used for ultrasonically actuated micro/nanoswimmers,^46–50^ a detailed system-level analysis and understanding of the coupled acoustic-structure dynamics involved in such acoustofluidic channels is missing.

Here, we present a flexural wave-driven acoustophoretic device for wall trapping of particles and cells. Under the excitation of microscope glass slide at its resonant frequencies, flexural waves can transfer the acoustic energy to the microfluidic channel. We utilize this phenomenon for trapping the particles and cells at the soft PDMS microchannel walls. Using numerical simulations and experiments, we show the underlying physical conditions for the wall trapping to happen in both rectangular and circular cross-sectional channels of different sizes. Finally, we demonstrate the interaction of motile cells (*e.g*., microalgae) with the soft walls under the ultrasonic forces. Our simulation and experimental results provide a method for the design of simple flexural-wave based acoustofluidic systems with the capability of wall trapping of microparticles/cells.

## Working principle

The model of a typical acoustofluidic device consists of a microchannel embedded in the solid substrate. For the microchannel actuation, solid substrates like glass or silicon are used as an acoustic waveguide driven by the piezoelectric transducer. In our acoustofluidic device, a piezo-transducer and a PDMS microchannel are attached on a rectangular thin glass plate, where the flexural modes of the glass plate are driven for the microchannel actuation. In previous acoustofluidic applications, acoustic waves like SAWs and BAWs can be modeled as the displacement boundary condition by a sinusoidal function in order to generate the simplified 2D numerical model.^21,23,29,35^ Therefore, we define the flexural waves as the sinusoidal function, which is applied on a glass substrate attached to the PDMS microchannel for the 2D numerical model. This method replaces the piezo transducer and the glass plate with the simplified displacement condition. The glass substrate and the PDMS container are defined as isotropic elastic solids. Further, the acoustic fields in the water channel are calculated by the first and second order perturbation equations as derived in ref. 35. First, the thermoacoustic equations are solved for the first order acoustic fields and thermoviscous boundary layer is calculated to determine the streaming effect near the walls. Second, the time-averaged second-order equations are solved by the resulting first-order fields in order to calculate the streaming force acting on particles. According to the assumptions explained above, we set the governing equations for the fields and the boundary conditions of the PDMS microchannel as derived previously.^35,51,52^ Furthermore, the acoustophoretic forces on microparticles suspended in the fluid are calculated from the governing fields and the particle tracing model is established at the end.

### Governing equations

In the typical acoustofluidic device driven by the Lamb waves, the microchannel is bonded on the solid substrate and aligned with the piezoelectric transducer as shown in ESI† Fig. S1a. For the actuation of microchannel, the Lamb waves propagate in the solid acoustic waveguide continuously and the whole device starts to vibrate at the corresponding frequency, as the sinusoidal voltage is applied on the electrodes of piezoelectric transducer. The Lamb waves consist of symmetric (longitudinal) and anti-symmetric (flexural) waves.^53^ The particle motions of the flexural and longitudinal waves are transverse and parallel to the direction of the wave propagation in solid plates, respectively. In our microfluidic device, the normal actuation of microchannel is applied on the bottom wall by the flexural waves. Therefore, we embedded the microchannel at the bottom center of the PDMS to utilize the flexural waves as shown in ESI† Fig. S1, where a thin glass plate is used as an acoustic waveguide. The governing equation of flexural waves in a thin plate is derived as:^54^

(1) $$\begin{array}{l} D\Delta^{2}W+\omega^{2}\rho hW=0,\\ D=\frac{Eh^{3}}{12\left(1-\nu^{2}\right)} \end{array}$$

where *E*, *h*, *ρ*, *ν*, *D* and Δ denote the elasticity modulus, thickness, density, Poisson’s ratio, flexural rigidity and Laplace operator, respectively. *W*(*x*, *z*) indicates the modes of the vibration where *w*(*x*, *z*, t) = *W*(*x*, *z*)e^*iωt*^. Eqn (1) shows the Kirchhoff–Love plate equation for the free harmonic vibration of thin plates, which is applicable when the plate length (*L*) is relatively larger than the plate thickness $\left(\frac{L}{h}\geq 10\right)$. The exact solution of eqn (1) gives the mode function and natural frequency for a thin plate with simply supported edges as:^55^

(2) $$\begin{array}{l} W_{m,n}=\sum_{m=1}^{\infty }\sum_{n=1}^{\infty }C_{mn}\sin\frac{m\pi x}{a}\sin\frac{n\pi z}{b}\\ \omega_{mn}=k^{2}\sqrt{\frac{D}{\rho h}},\;k^{2}={\left(k_{1}\right)}^{2}+{\left(k_{2}\right)}^{2} \end{array}$$

where the simply supported boundary conditions are defined as:

(3) $$\begin{array}{l} \frac{\partial^{2}w}{\partial x^{2}}+\nu \frac{\partial^{2}w}{\partial z^{2}}\;\text{for}\;x=0\;\text{and}\;x=a\\ \nu \frac{\partial^{2}w}{\partial x^{2}}+\frac{\partial^{2}w}{\partial z^{2}}\;\text{for}\;z=0\;\text{and}\;z=b \end{array}$$

where *a* and *b* are the plate dimensions. *C_mn_* is the vibration amplitude for each value of *m* and *n*; *k* denotes the total wavenumber for flexural waves in both *x* and *z* dimensions where *k*_1_ = *mπ*/*a*, *k*_2_ = *nπ*/*b*. The natural frequency of each mode can be obtained with corresponding *m* and *n* value. In our 2D numerical model, the flexural waves are defined by the sinusoidal function as a simplified form of eqn (2), that will be explained in the next subsections.

The glass plate and PDMS cover are defined as the isotropic elastic material. The governing equation for the displacement field *u* in an elastic solid with the density of *ρ_s_* is given by the Cauchy equation as below:

(4) $$-\rho_{\text{s}}\omega^{2}u=\nabla \cdot \sigma$$

where the linear stress–strain relation is given in terms of the elastic moduli *C*_ik_ by the Voigt representation as follows:

(5) $$\left(\begin{array}{l} \sigma_{xx}\\ \sigma_{yy}\\ \sigma_{zz}\\ \sigma_{yz}\\ \sigma_{xz}\\ \sigma_{xy} \end{array}\right)=\left(\begin{array}{cccccc} C_{11} & C_{12} & C_{12} & 0 & 0 & 0\\ C_{12} & C_{11} & C_{12} & 0 & 0 & 0\\ C_{12} & C_{12} & C_{11} & 0 & 0 & 0\\ 0 & 0 & 0 & C_{44} & 0 & 0\\ 0 & 0 & 0 & 0 & C_{44} & 0\\ 0 & 0 & 0 & 0 & 0 & C_{44} \end{array}\right)\left(\begin{array}{c} \partial_{x}u_{x}\\ \partial_{y}u_{y}\\ \partial_{z}u_{z}\\ \partial_{y}u_{z}+\partial_{z}u_{y}\\ \partial_{x}u_{z}+\partial_{z}u_{x}\\ \partial_{x}u_{y}+\partial_{y}u_{x} \end{array}\right)$$

For an isotropic material, the elastic moduli *C*_11_, *C*_12_ and *C*_44_ are characterized by the longitudinal sound speed (*c*_lo_) and the transverse sound speed (*c*_tr_); *C*_11_ equals to $c_{\text{lo}}^{2}\rho_{\text{s}}$ and *C*_44_ equals to $c_{\text{t}\dot{\text{r}}}^{2}\rho_{\text{s}}$, where *C*_12_ = *C*_11_ − 2*C*_44_. The damping coefficient is negligible for the glass plate (*Γ* = 0.0004), whereas the PDMS is a soft material with high damping. Further, the transverse and longitudinal characteristics of PDMS are very different, so the damping must be defined for the elastic moduli, separately. Therefore, the damping coefficient of the PDMS container is defined by the complexvalued elastic moduli as presented in Table 1.

For the fluid domain, the governing perturbation equations for the thermoacoustic fields are derived as in ref. 61, where the harmonic time dependence of all fields is treated as *δ*_t_ → −*iω*. Thus, the heat transfer equation for the first order temperature field *T*_1_, the kinematic continuity equation in terms of the first order pressure field *p*_1_, and the dynamic Navier–Stokes equation for the first order velocity field ***ν***_1_ are given by

(6) $$\begin{array}{l} i\omega T_{1}=i\omega \frac{\alpha T_{\text{f}}}{\rho_{\text{f}}C_{\text{p}}}p_{1}-D_{\text{th}}\nabla^{2}T_{1}\\ i\omega p_{1}=\frac{1}{\gamma \kappa_{\text{f}}}\left[i\omega \alpha T_{1}+\nabla \cdot \nu_{1}\right]\\ \dot{\iota }\omega \rho_{\text{f}}\nu_{1}=\nabla p_{1}-\eta \nabla^{2}\nu_{1}-\beta \eta \nabla \left(\nabla \cdot \nu_{1}\right) \end{array}$$

where *D*_th_, *γ*, *α*, *η*, *C*_p_ and *ω* denote the thermal diffusivity, specific heat capacity ratio, thermal expansion coefficient, viscosity, specific heat capacity and angular frequency, respectively. The temperature of fluid (*T*_f_) equals to 25 °C before the presence of acoustic wave. *ρ*_f_ is the density of fluid and *β* is the viscosity ratio. Further, solutions of eqn (6) give the viscous boundary layer thickness (*δ*), which equals to ${\left(\frac{2\eta }{\rho_{\text{f}}\omega }\right)}^{0.5}$. For acoustic waves at 100 kHz in the water, the viscous boundary layer thickness is calculated as 1.69 μm, which indicates that the Rayleigh–Schlichting boundary-layer theory for the acoustic streaming is valid for our acoustofluidic setups, where *δ* ≪ *H*_ch_ ≪ *λ*.^62^ *H*_ch_ denotes the height of the channel. In other words, the streaming effect near the walls generate vortexes inside the channel, that may affect the wall trapping of microparticles/cells. Therefore, we included the streaming force by the second order perturbation equations in our calculations.

In the first order equations, the temperature field has little effect and can be negligible as reported in ref. 35, where *η* and *β* are constants. So, we used the second-order equations as derived in ref. 51, where the thermal effect is neglected. Assuming harmonic time dependence, the time average of the second-order continuity and Navier–Stokes equation are given by

(7) $$\begin{array}{l} \rho_{\text{f}}\nabla \cdot \langle \nu_{2}\rangle =-\nabla \cdot \langle \rho_{1}\nu_{1}\rangle \\ \eta \nabla^{2}\langle \nu_{2}\rangle +\beta \eta \nabla \left(\nabla \cdot \langle \nu_{2}\rangle \right)-\langle \nabla p_{2}\rangle =\langle \rho_{1}\delta_{\text{t}}\nu_{1}\rangle +\rho_{\text{f}}\langle \left(\nu_{1}\cdot \nabla \right)\nu_{1}\rangle \end{array}$$

where 〈***ν***_2_〉 denotes the streaming velocity. In the 2D numerical model, the computational domains are defined by the governing equations as presented above and we illustrate the boundary conditions in the following section.

### Boundary conditions

Four boundary conditions are defined for the acoustofluidic device. First, the actuation of the microchannel is represented by a given displacement in the normal direction on the PDMS bottom wall where the glass plate is attached. Second and third boundary conditions represent the interaction between the fluid and solid domains, where the stress and the velocity fields are continuous at all fluid–solid interfaces. Finally, the stress is zero on all air–solid interfaces. Therefore, the displacement actuation of microchannel, the interactions of fluid–solid interface and air–solid interface are given as

(8) $$\begin{array}{ll} \text{solid}\;\text{domain}\;\leftarrow \;\text{displacement}\;\text{actuation:} & u=\pm d_{0}n\\ \text{fluid}\;\text{domain}\;\leftarrow \;\text{solid}\;\text{domain:} & \nu \cdot n=-i\omega u\cdot n\\ \text{solid}\;\text{domain}\;\leftarrow \;\text{fluid}\;\text{domain:} & \sigma \cdot n=-pn\\ \text{solid}\;\text{domain}\;\leftarrow \;\text{air:} & \sigma \cdot n=0 \end{array}$$

where *d*_0_ indicates the amplitude of displacement actuation.

### Model system and computational domain

In this part, we present the 2D numerical model for the acoustofluidic device and explain the implementation of the governing equations for the model system using the finite element method by the COMSOL Multiphysics 5.4. The typical acoustofluidic device for the particle manipulation involves multi-physics problems that consist of electromagnetics for the piezo material, solid-structure for the solid domains and fluid dynamics for the fluid domain. The most of the acoustofluidic systems are constructed as simplified 2D models by the physical approaches, because the modeling that covers all physics is too complex.^21,23,29,35^ Thus, we designed our acoustofluidic system as a 2D numerical model. The dimensions of the 2D numerical model are listed in Table 2. The microfluidic channels are designed as rectangular and circular channels which are located at the center bottom of the PDMS substrate as shown in ESI† Fig. S1b. In this work, we used several rectangular microchannels of 100 μm, 200 μm, 500 μm, and 750 μm width (*W*_ch_) and 100 μm height (*H*_ch_). Further, we used circular channels of 200 μm, 300 μm, 400 μm and 500 μm diameter (*D*_ch_). The distances of channels from the bottom PDMS layer (*D*_bottom_) equal to 0 and 100 μm for the rectangular and circular microchannels, respectively. The length of the PDMS channel (L) is 8.67 mm, where *H*_PDMS_ = 4 mm and *W*_PDMS_ = 6 mm. We designed the height (*H*_PDMS_) and the width (WPDMS) of the PDMS container as relatively large in order to absorb most of the acoustic waves coming from the glass plate and the water channel.

The boundary conditions and computational domains for the 2D numerical model of the acoustofluidic system are illustrated in the ESI† Fig. S2. First, we model the displacement actuation for the *x*–*y* plane cross section of glass plate. In the modal analysis of the device, it is illustrated that the flexural modes of whole model have little changes in resonant frequencies compared to the flexural modes of a plate as shown in ESI† Fig. S3. According to the results of the analysis, the displacement profile applied on the microchannel can be obtained by the modes of vibration *W*(*x*, *z*) for the plate instead of analyzing the whole model. In this study, the microparticles are manipulated in the *x*–*y* plane, thus we consider the flexural modes on *x* dimension and neglect the flexural modes on *z* dimension of the glass plate. Therefore, *W*(*x*, *z*) given in eqn (2) is simplified as sin(*k*_1_*x*) with the amplitude *C_m_* for the *x*–*y* plane cross section of glass plate and then, we apply the sinusoidal function of *C_m_* sin(*k*_1_*x*) on the glass plate for the displacement actuation of PDMS microchannel as shown in ESI† Fig. S4. This sinusoidal function is also equal to the amplitude of displacement actuation (*d*_0_) presented in eqn (8).

In ESI† Fig. S4, the displacement profile of flexural waves in the *x*–*y* plane is applied on the bottom of PDMS channel as sinusoidal functions by a glass substrate. Two types of displacement profile, which are sine and cosine waves, are applied for the implementation of pressure distribution inside the water channel. The cosine-waveform type displacement is used to align the pressure antinode at the center of PDMS channel, where the sine-waveform type displacement is used to align the pressure node at the center of PDMS channel as shown in ESI† Fig. S4a and b, respectively. By aligning the pressure distribution inside the channel, one can generate the acoustophoretic motion of particles through the pressure nodes.^52^ Further, the acoustic fields inside the water channel are calculated numerically with the thermoviscous acoustics and laminar flow interfaces by the COMSOL Multiphysics software. The thermoviscous acoustics interface is used to solve the first order perturbation equations as presented in eqn (6) and the laminar flow interface is used to solve the second order perturbation equations as presented in eqn (7). Then, the solid mechanics interface is used to solve the governing equation presented in eqn (4) for the glass plate and PDMS container. Moreover, the boundary conditions between fluid–solid interfaces are defined by the multiphysics interface between the thermoviscous acoustics and solid mechanics interfaces. Finally, the acoustic fields of the fluid gives the acoustic radiation and streaming forces acting on the particles and then, the particle motion can be tracked for the suspended particles as described in the following section.

### Particle tracing model

The acoustophoretic motion of microparticles is modeled by the particle tracing module in the COMSOL simulation tool. This module provides the particle motion where the spherical polystyrene particles with various diameters are uniformly distributed in the fluid channel and the forces acting on the particles are applied for the manipulation. In the 2D numerical model, the radiation force (***F***^rad^), the drag force (***F***^drag^) and the gravitational force (***F***^grav^) are considered, whereas the particle–particle interaction are neglected. The buoyancy force is included in the gravitational force $\left(F^{\text{grav}}=\frac{4}{3}\pi a^{3}\left(\rho_{\text{f}}-\rho_{\text{p}}\right)g\right)$. As the radius of spherical particle (*a*) is much smaller than the wavelength of acoustic waves (*λ*), the radiation force on the small particles is given by^52^ as below:

(9) Frad=−πa3[2κf3Re[f1∗p∗∇p]−ρfRe[f2∗ν∗⋅∇ν]]

In Table 2, the material properties for the particle and the fluid are indicated. $\kappa_{\text{f}}=1/\left(\rho_{0}c_{0}^{2}\right)$ is the compressibility of the fluid. Re and the asterisk (*) denote the real and the complex conjugate part of the parameter, respectively. The monopole coefficient *f*_1_ and the dipole coefficient *f*_2_ are given by

(10) $$f_{1}=1-\frac{\kappa_{\text{p}}}{\kappa_{\text{f}}}\text{and}f_{2}=\frac{2\left(\rho_{\text{p}}-\rho_{\text{f}}\right)}{2\rho_{\text{p}}-\rho_{\text{f}}}$$

where *κ*_p_ and *ρ*_p_ are the compressibility and the density of the particle, respectively. When the wall effect is negligible,^63^ the time-averaged Stokes drag force on a spherical particle is given by

(11) $$F^{\text{drag}}=6\pi \eta a\left(\langle \nu_{2}\rangle -\nu_{\text{p}}\right)$$

where *a* and ***ν***_**p**_ are radius and velocity of the particles, respectively. Then, the motion of the particle can be obtained by the Newton’s second law of motion

(12) $$m\frac{\text{d}\nu_{\text{p}}}{\text{d}t}=F^{\text{rad}}+F^{\text{drag}}+F^{\text{grav}}$$

where *m* is the mass of the particle. For many problems in microfluidics, the inertia of particles can be negligible, where the characteristic time of acceleration is so small compared to the time scale of the particle motion.^64^ This approach simplifies the eqn (12) as ***F***^drag^ = −***F***^rad^ − ***F***^grav^. So, the particle velocity is calculated as below

(13) $$\nu_{\text{p}}=\langle \nu_{2}\rangle +F^{\text{rad}}+\frac{F^{\text{grav}}}{6\pi \eta a}$$

In this paper, the particle trajectories are calculated as the streamlines of the particle velocity (***ν***_**p**_) for the steady flow.

## Results and discussion

### Flexural-wave based device design and fabrication

**Fig. 1:**
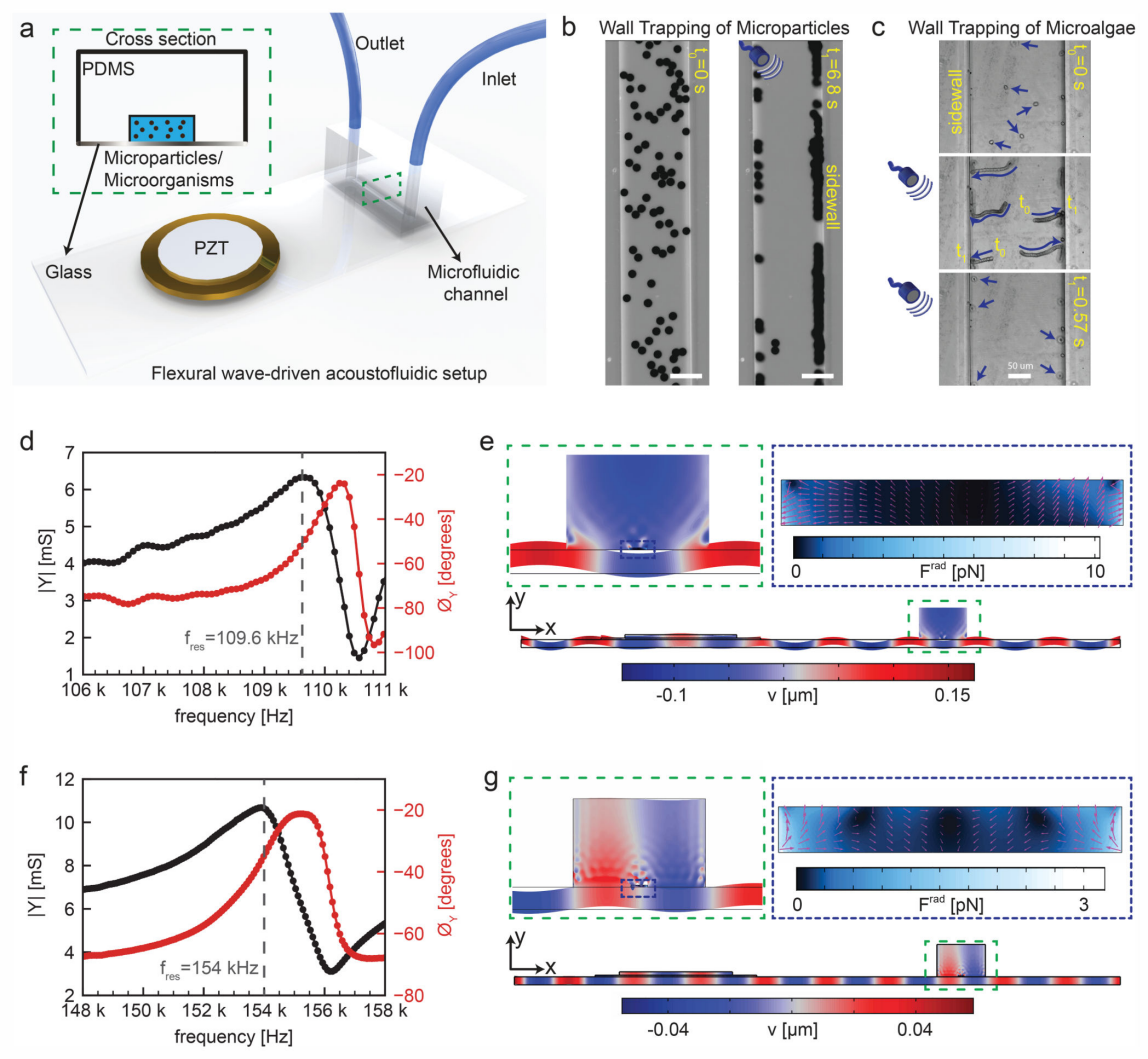
Working principles of the flexural-wave driven soft attractor walls. (a) Schematics of the experimental setup (b) wall trapping of 10 μm diameter polystyrene (PS) particles under ultrasound during 6.8 seconds (c) wall trapping of microalgae inside the rectangular channel under ultrasound during 0.57 seconds (d) admittance-phase plots of the system round 109.6 kHz resonance frequency corresponding to cosine-type displacement of the channel (e) numerical simulations of the complete setup at calculated resonance frequency of 103 kHz, the blue-red color bar indicates the transverse displacement in μm scale with a close-up in dashed green inset, and the dashed-blue inset shows the acoustic radiation force in pN range inside the rectangular channel, where the pink arrows show the direction of force (f) admittance-phase plots of the system round 154 kHz resonance frequency corresponding to sine-type displacement of the channel (g) numerical simulations of the complete setup at calculated resonance frequency of 103 kHz, the blue-red color bar indicates the transverse displacement in μm scale a close-up in dashed green inset, and the dashed-blue inset shows the acoustic radiation force in pN range inside the rectangular channel, where the pink arrows show the direction of force.

We fabricated the acoustofluidic channel using three main components: a piezoelectric transducer, a standard 1 mm thick microscope glass slide with 75 mm by 25 mm dimensions and a soft microchannel, as shown in Fig. 1a. The PDMS microfluidic channel was chosen as a reservoir for injection of microparticles or microorganisms. For microorganism candidate in this study, *Chlamydomonas reinhardtii* (*C*. *reinhardtii*) microalgae were selected as natural microswimmers. The example of acoustophoretic motion of 10 μm polystyrene particles and microalgae towards the soft boundaries are shown in Fig. 1b and c. Once the dispersed microparticles/microalgae are exposed to ultrasonic waves at certain frequencies, the acoustic radiation force can push them towards the PDMS wall for the reasons that will be explained fully in the following sections. After assembling the flexural wave-driven setup, we characterized the resonance frequencies of the system through electromechanical impedance measurements (details in the Materials and methods section). Two representative resonance frequencies are shown *via* admittance amplitude and phase curves in Fig. 1d and f. The positions of the piezoelectric transducer and microchannel were optimized through finite-element simulations for maximum acoustic energy transfer. There are two distinctive actuation modes in which the flexural waves periodically deform the microchannel. In the first case shown in Fig. 1d and e, the peak of flexural wave hits the center of microchannel, representing a cosine-waveform type displacement. Whereas, in the second case shown in Fig. 1f and g, the nodal line of flexural wave is present under the center of microchannel, representing a sinusoidal-waveform type displacement. The first actuation mode creates the maximum acoustic pressure amplitude at the center of microchannel, hence creating the acoustic radiation force with a direction from the center towards the sidewalls. However, the second actuation mode puts the minimum pressure amplitude at the center of microchannel, thereby directing the acoustic radiation force towards the center. For the purpose of this study, we focus on the first actuation mode which hereafter we refer to as wall trapping.

We investigated the location of the piezoelectric transducer on the glass substrate, as shown in Fig. S5.† The location of the transducer alters the resonance frequency of the whole system slightly due to the change in the modal mass and modal stiffness. We found that, for a given flexural mode, the maximum energy transfer and hence radiation force occurs when the PZT is mounted away from the edge of the glass substrate by its one diameter (20 mm in this case). Note that, the observed wall trapping effect remains universal, and only the magnitude of radiation force would change. Further, we characterized the location of the PDMS channel for the piezoelectric transducer/glass plate configuration mentioned above, as shown in ESI† Fig. S6. The change in the resonant frequency of the whole system is insignificant, while the PDMS channel is shifted on the glass plate for the corresponding flexural mode. We found that both cosine- and sine-like deformation of PDMS channel can be carried out by moving the channel and aligning it on maximum- or zero-amplitude bending mode of the system.

### Rectangular cross-section channels

**Fig. 2:**
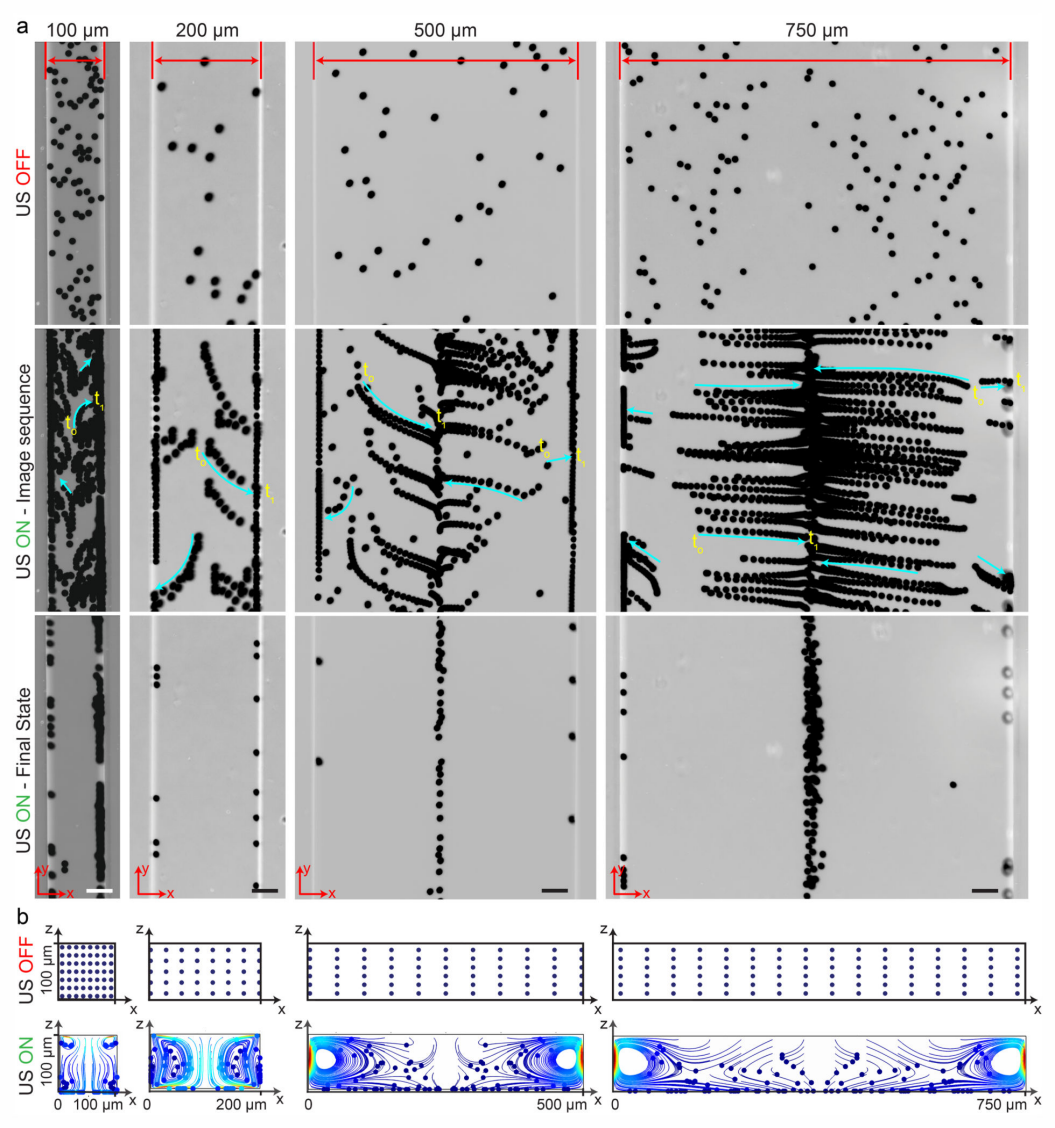
Wall trapping of 10 μm PS particles in different rectangular microchannels. (a) Time-lapse images of particles under ultrasound. The narrower 100 μm and 200 μm width channels show excellent wall trapping, whereas the wider 500 μm and 750 μm width channels have poor wall-trapping performance due to the sine-type displacement actuation. (b) The particle tracing simulations of the microchannel cross-section from dispersed to ultrasonically manipulated condition over 15 seconds, confirming the final location of the beads in the experiments (Movie S1†). The blue and red colors indicated the minimum and maximum speed of the particles, respectively. The scale bars are 50 μm. The operating frequencies are 105 kHz for 100 μm and 200 μm width channels and 155 kHz for 500 μm and 750 μm width channels.

Here, we investigated the performance of rectangular channels in wall trapping of microparticles. We fabricated four different microchannels of 100 μm, 200 μm, 500 μm, and 750 μm width and 100 μm height. The reason behind the various designs is that the acoustic wavelength is much larger than the channel width at the excitation frequencies below 200 kHz. This means that for frequencies *f* < 200 kHz, the standing waves in the quartz glass would have a wavelength of 6 ⪅ λ ⪅ 14 mm for operating frequency of 50 kHz < *f* < 200 kHz (ESI† Note S1 and Fig. S10). Fig. 2a presents the acoustophoretic motion of 10 μm PS particles for different actuation modes for different microchannel widths (Movie S1†). We found that for microchannels of 100 and 200 μm width, the wall trapping mode was prevalent among different resonance frequencies of the glass substrate. On the other hand, the microchannels of 500 and 750 μm width showed different trapping patterns, which is attributed to sinusoid-like displacement waveform as depicted in Fig. 1g. To further understand this behaviour, we preformed two-dimensional particle tracing simulations for the cross-section of the microchannels, as shown in Fig. 2b. The displacement profiles, cosine-type for 100 and 200 μm and sinusoid-type for 500 and 750 μm channels, with the same excitation frequencies from the experiments were applied to verify the particles behaviour. It is clear the wall trapping occurs for all channels with microparticles trapped in the sidewalls. However, the wider channels also show center trapping of microparticles which is attributed to nodal line in the acoustic pressure field. This may be not favourable for homogeneous wall-trapping purpose since a large number of particles/cells would be trapped in the center rather than the sidewalls of the channel.

For wall trapping manipulation in a PDMS channel, the microfluidic device has a well-defined cosine-waveform type displacement for the operating frequency of around 103 kHz according to Fig. 1e. Therefore, we performed the characterization of PDMS height (*H*_PDMS_) and PDMS width (*W*_PDMS_) for the microfluidic device with the operating frequency range of 102 to 104 kHz, as shown in ESI† Fig. S7 and S8, respectively. The simulations show that the maximum radiation force shifts by the change in the width and height of the PDMS channel due to the slight shift of the resonance frequency of the whole system. For this, we computed the frequency dependency of the spatially averaged radiation force $\left(\bar{F^{\text{rad}}}\right)$ for 10 μm particles in the fluid domain:

(14) $$\begin{array}{l} \bar{F_{x}^{\text{rad}}}=\frac{1}{W_{\text{ch}}H_{\text{ch}}}\int_{\Omega_{\text{ch}}}\frac{x}{\left|x\right|}F_{x}^{\text{rad}}\text{d}x\text{dy},\\ \bar{F_{y}^{\text{rad}}}=\frac{1}{W_{\text{ch}}H_{\text{ch}}}\int_{\Omega_{\text{ch}}}\left|F_{y}^{\text{rad}}\right|\text{d}x\text{d}y \end{array}$$

where we rectified the *x*-component of the spatially averaged radiation force $\left(\bar{F_{x}^{\text{rad}}}\right)$ by the prefactor of $\frac{x}{\left|x\right|}$. This is for identifying the resonances which have similar behavior with the wall trapping, where the positive $\bar{F_{x}^{\text{rad}}}$ indicates the average acoustic radiation force with the direction from the center towards the wall of the channel. It is important to note that the bottom center of the channel is placed at the origin in simulations. We also took the absolute value of $F_{y}^{\text{rad}}$ before averaging in order to increase the effect of vertical acoustic radiation forces, while $F_{y}^{\text{rad}}$ can be too small to identify the resonances for the wall trapping application. In ESI† Fig. S7 and S8, the heat maps of $\bar{F_{x}^{\text{rad}}}$ and $\bar{F_{y}^{\text{rad}}}$ are presented for the system operating around 103 kHz with the different PDMS heights and widths, respectively. It is worth noting that, in the experiments, the frequency tuning is straightforward which makes the wall trapping robust to changes in the PDMS block size.

Furthermore, we illustrated the importance of the ratio of wavelengths (*λ*) to channel width (*W*_ch_) at lower frequencies (<200 kHz) for the wall trapping applications, where the simplified 2D model is used to implement the corresponding cosine-type displacement, as shown in ESI† Fig. S9. We found that the larger wavelength/channel-width ratio results in a much higher radiation force in both *x* and *y* directions, which leads to an increase in the wall trapping effect. Though, at small *λ*/*W*_ch_ ratios, the pressure nodes appear inside the channel, which causes a poor wall trapping performance as the microparticles are collected at the nodal points. For the microchannel sizes and excitation frequencies used in the experiments, the acoustic wavelength/ channel-width ratio ranges between 10–150.

**Fig. 3:**
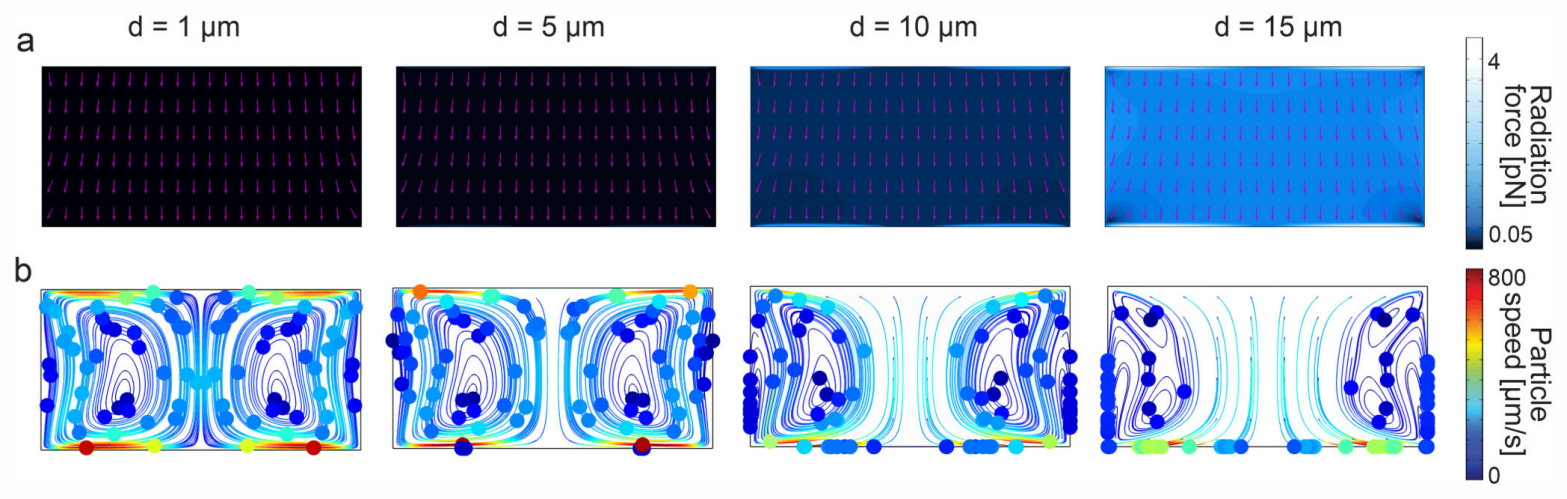
Analysis of wall trapping strength for different particle diameters in the rectangular cross-sectional channels. (a) The acoustic radiation force shows a significant increase for the increasing particle diameters. The purple arrows indicate the direction of radiation forces. (b) The particle tracing simulations over 25 seconds by accounting both acoustic radiation and streaming forces; the strong wall trapping begins for particles approximately larger than 10 μm diameter, whereas the smaller ones follow the streaming-induce vortex. The channel width is set to 200 μm, where the operating frequency is 105 kHz. The displacement of 1 × 10^−7^ cos(*kx*) is applied on the bottom PDMS layer.

To further understand the underlying forces behind the acoustophoretic motion of particles in such flexural-wave based channel, we need to distinguish the roles of the acoustic radiation and streaming forces. The scaling laws in acoustophoresis indicate that acoustic radiation force is volume dependent *F*^rad^ ∝ *a*^3^, whereas the streaming induced drag force is radius dependent *F*^drag^ ∝ *a*.^65^ In order to find the dominant acoustic force, we performed FE simulations for different particle sizes as shown in Fig. 3. The numerical results show that for particles with diameter bigger than 10 μm the radiation force becomes stronger, as shown in Fig. 3a, hence enables the wall-trapping mode, as shown in Fig. 3b; whereas, the smaller-diameter particles follow the acoustic streaming-induced vortices. The extra information regarding the displacement profile of the bulk PDMS, first-order pressure and velocity, and second-order streaming velocity inside the microchannel is given in ESI† Fig. S11.

The cross over from radiation force dominated particle motion to acoustic streaming-induced drag force dominated particle motion is determined theoretically by calculating the critical particle diameter (*d*_c_).^35^ The critical particle diameter is derived by equating two forces (*F*^rad^ = *F*^drag^), which results in the following equation:

(15) $$d_{\text{c}}=\sqrt{12\frac{\psi }{\phi }}\delta \approx 8.9\mu \text{m}$$

where *ϕ* is the acoustophoretic contrast factor defined as:

(16) $$\phi (\tilde{\kappa },\tilde{\rho })=\frac{1}{3}\left[\frac{5\tilde{\rho }-2}{2\tilde{\rho }+1}-\tilde{\kappa }\right]$$

(17) $$\tilde{\rho }=\frac{\rho_{\text{p}}}{\rho_{\text{f}}},\tilde{\kappa }=\frac{\kappa_{\text{p}}}{\kappa_{\text{f}}}$$

where *ψ* is the geometry dependent factor of order unity. We decided the *ψ* value based on the previous calculation for a standing wave parallel to a planar wall, which is defined for Rayleigh streaming.^35,65,66^ Muller *et al*.^35^ used the same geometry-dependent factor (*ψ* = 3/8) as a reference value to find the critical particle diameter in different geometries, where they found a slight deviation in the *ψ* value according to their geometry design. Besides, finding the accurate geometry-dependent factor requires a parameter fitting with precise experiments by varying the particle size, medium type, excitation frequency, and channel geometry. However, this was not the scope of this paper and would be the subject of another study. The acoustic contrast factor *ϕ* is calculated as 0.163 for the polystyrene microparticles by using the material parameters presented in Table 1. The viscous boundary layer thickness is calculated as 1.69 μm for the acoustic waves at 100 kHz in the water, leading to the approximate critical particle diameter of 8.9 μm, which is close to the wall trapping cases in simulations and experiments.

### Circular cross-section channels

**Fig. 4:**
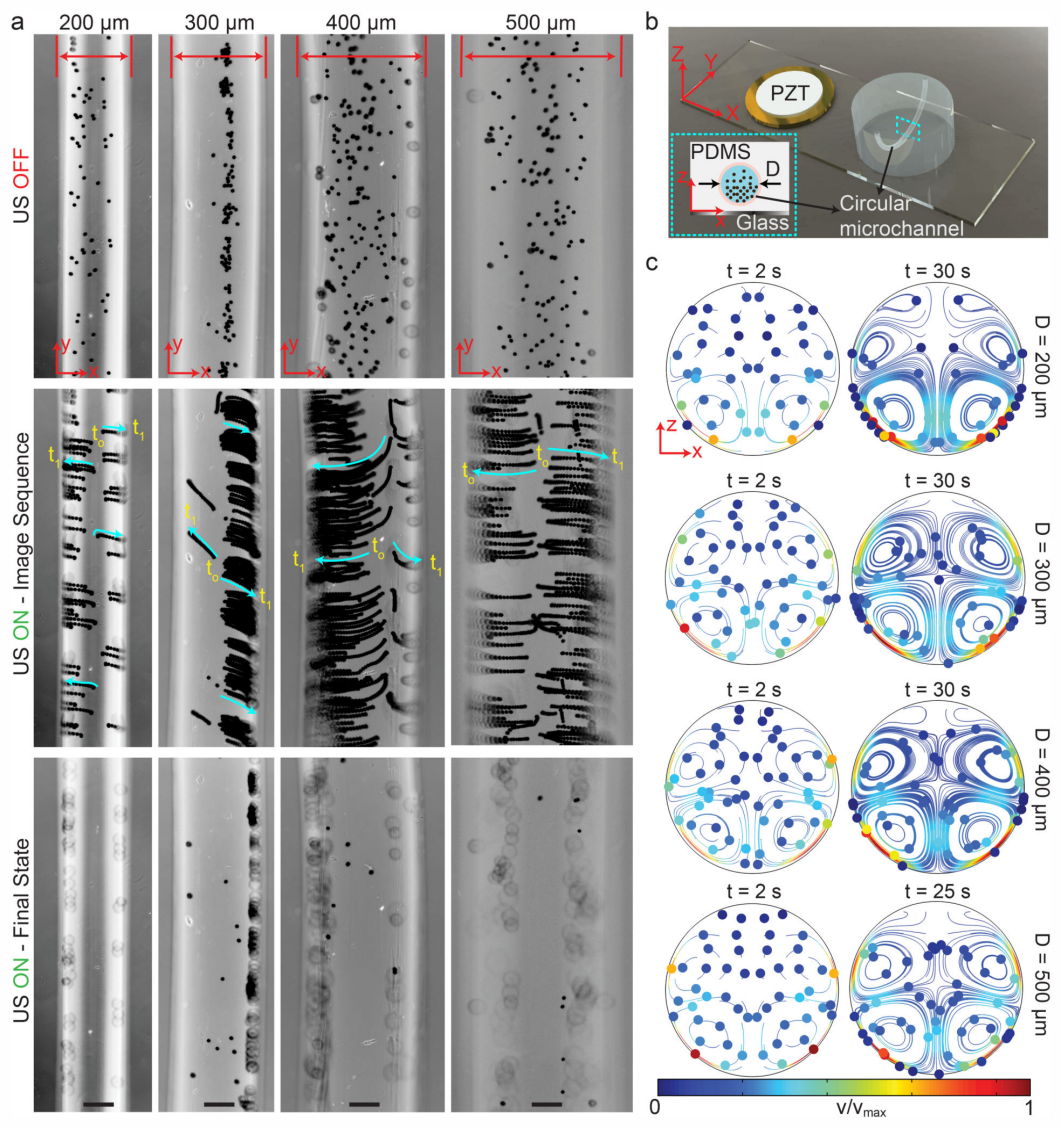
Wall trapping of 10 μm PS particles in different capillary channels. (a) Time-lapse images of particles under ultrasound for 200 μm to 500 μm diameter channels (Movie S2†) (b) schematics of the acoustofluidic setup for wall trapping inside artificial capillaries. (c) The particle tracing simulations of the microchannel cross-section from dispersed to ultrasonically manipulated condition. The traces confirm the final location of the beads observed in the experiments. The scale bars are 100 μm. The operating frequencies are 87 kHz for 200 μm diameter channel, 47 kHz for 300 μm diameter channel, 46.8 kHz for 400 μm diameter channel and 32.9 kHz for 500 μm diameter channel.

We fabricated the circular-channel acoustofluidic setup similar to previous rectangular cases with different diameters, as shown schematically in Fig. 4b. The three-dimensional circular channel represents the biologically relevant capillaries, which is a useful platform for understanding the global acoustophoretic motion of particles/cells near the vessel walls. To achieve wall trapping of particles, we excited the system at resonance frequencies below 100 kHz. It is important to note that since the whole microchannel is surrounded by the lossy PDMS material, a larger displacement from the glass substrate is required to generate sufficient acoustic radiation force. Fig. 4a shows the time-lapse images from the start to final positions of 10 μm during the ultrasonic actuation (Movie S2†). During the wall-trapping stage, the microparticles migrated from the bottom of channel at the focal plane of the microscope towards the curved sidewalls at higher heights, as shown in the last row of Fig. 4a. We could verify this climbing motion of the particles through numerical simulations presented in Fig. 4c. During the 30 seconds for 200 μm channel, and 60 seconds for 300, 400, and 500 μm channels, the particle tracing result shows the trapping of particles in the sidewalls. It’s worth noting that, in the simulations, the particles are initially dispersed homogeneously, which is why we also see the vortices at top-half of the channel. But, of course in the experiments, the particles were all sedimented by gravity, so we could not observe any motion at the top part of the channels.

**Fig. 5:**
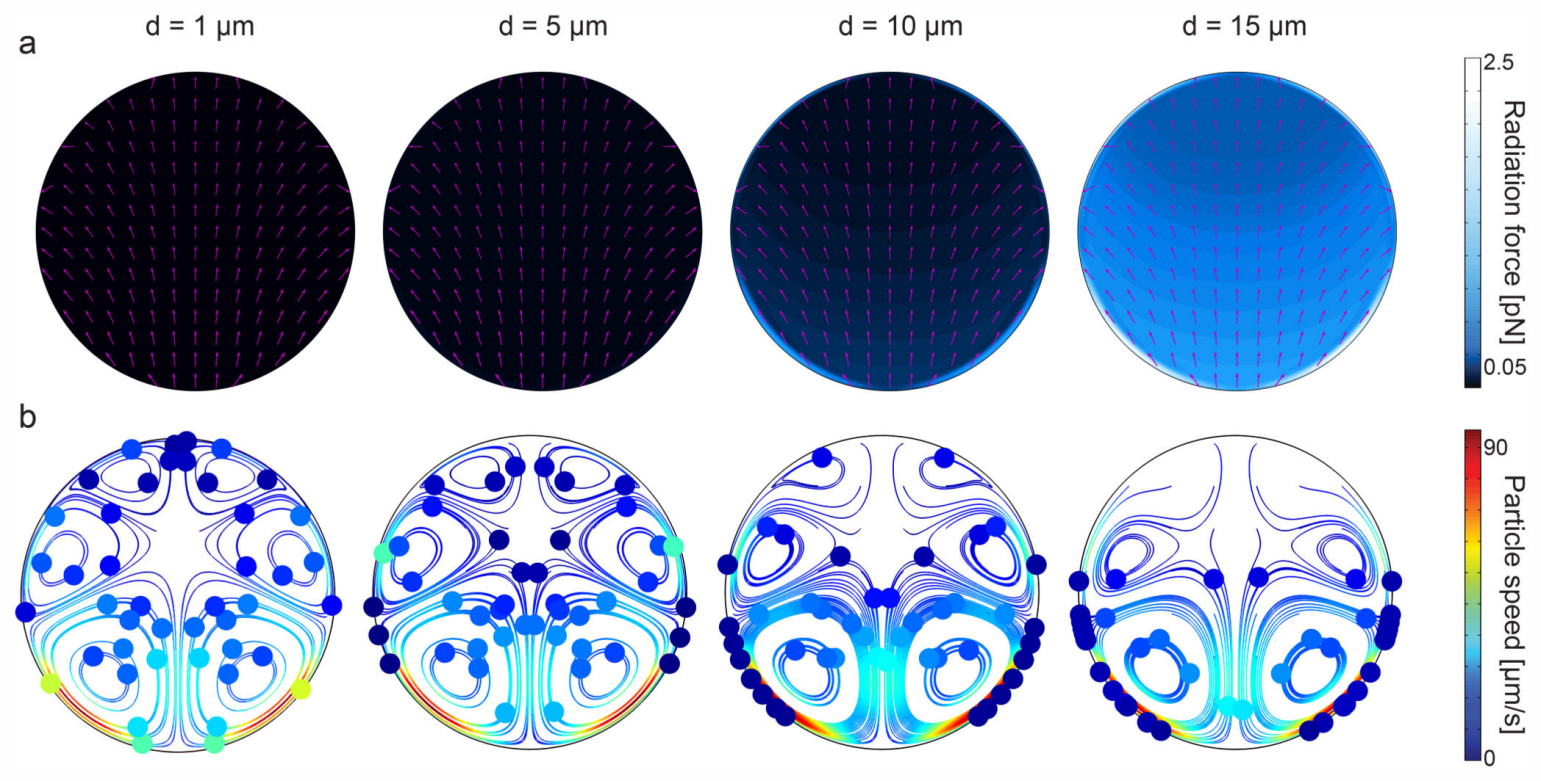
Analysis of wall trapping strength for different particle diameters in the circular cross-sectional channels. (a) The acoustic radiation force shows a significant increase for the increasing particle diameters. The purple arrows indicate the direction of radiation forces. (b) The particle tracing simulations over 27 seconds by accounting both acoustic radiation and streaming forces; the strong wall trapping begins for particles approximately larger than 10 μm diameter, whereas the smaller ones follow the streaming-induce vortex. The channel width is set to 200 μm, where the operating frequency is 87 kHz. The displacement of 1 × 10^−7^ cos(*kx*) is applied on the bottom PDMS layer.

Similar to the rectangular channels, we computed the acoustic radiation force for 1, 5, 10, and 15 μm particle diameters, shown in Fig. 5a. Noticeably, the maximum radiation force magnitude is much lower than the one in the rectangular channel, given the same displacement amplitude. This is attributed to the damped elastic waves in the PDMS medium, which is between the glass substrate and the circular channel. As a result of higher radiation force for larger-diameter particles, we find that 1 μm particles are following the streamlines, whereas, the particles bigger than approximately 10 μm diameter are trapped to the sidewalls. The extra information regarding the displacement profile of the bulk PDMS, first-order pressure and velocity, and second-order streaming velocity inside the microchannel is given in ESI† Fig. S12. It is worth recalling that, in contrast to soft circular PDMS channels, the particle focusing at the center of channel is possible in glass capillaries (*i.e*., hard boundaries) using bulk acoustic waves.^67–69^

### Cell-wall interaction

Single-cell manipulation is an important technique for biomedical applications, such as drug delivery and microsurgery.^70,71^ So far, the majority of the single-cell manipulation research has been focused on non-swimming cells. However, trapping motile organisms is challenging, due to their high velocities.^72^ Previously, it has been demonstrated that optical tweezers can be utilized for trapping and manipulation of motile microorganisms, including flagellated bacteria. Nevertheless, the optical tweezers require high-power lasers for the manipulation, which might be detrimental for cells. The other methods that can be applied to trap and manipulate motile microorganisms include magnetic tweezers^73^ and optically induced dielectrophoresis systems,^2^ which require additional experimental steps, such as magnetic labeling and photoconductor substrate usage to create a nonuniform electric field, respectively. Recently, the acoustic field is used to capture highly motile microalgae cells for harvesting, which is an important step in biofuel production process.^74,75^

**Fig. 6:**
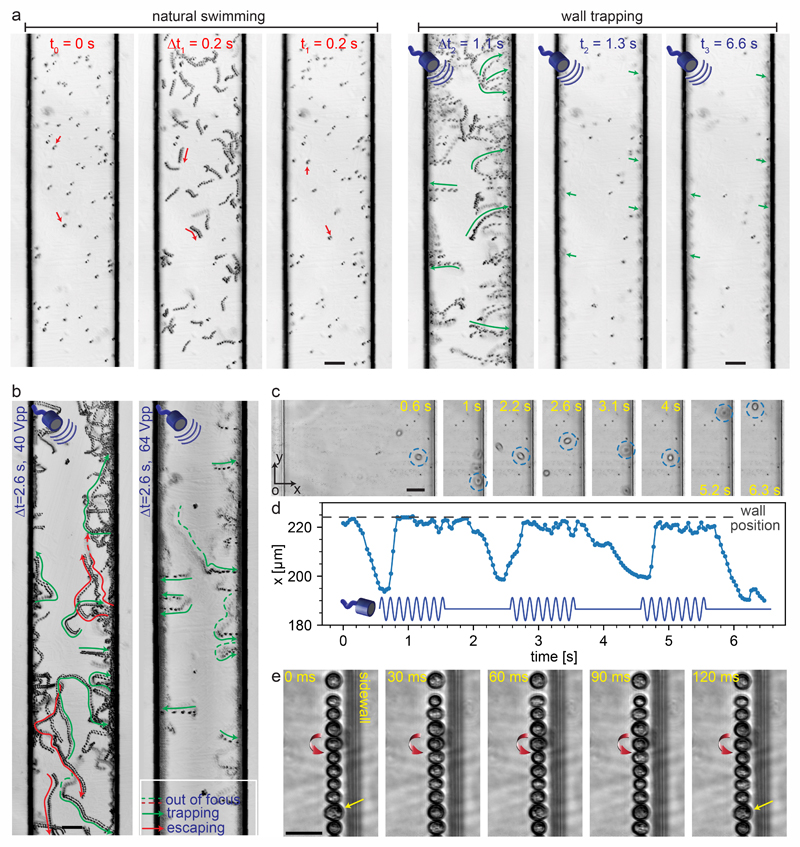
Microalgae-wall interactions under ultrasonic trapping forces. (a) Time-lapse images show trajectory of microalgae during 0.2 seconds natural swimming interval to the wall trapping stage during the next 6.4 seconds (Movie S3†). (b) The effect of the driving voltage amplitude on the trapping of microalgae swimmers, the green and red arrows show the trapping and escaping trajectories, respectively (Movie S4†). (c) The time-lapse images of the single microalgae under pulsed ultrasound signal of 2 seconds width, with its center position in *x*-axis plotted in (d) (Movie S5†). (e) The rotational behavior of the microalgae at the wall boundaries, with a direction denoted by red arrows (Movie S6†). The scale bars in (a) and (b) are 50 μm, and in (c) and (e) are 25 μm.

In the present study, we selected unicellular green microalgae, *Chlamydomonas reinhardtii* (*C. reinhardtii*) cells, for testing the wall-trapping capability of our acoustofluidic device. Microalgae are natural microswimmers with size range relevant to acoustic radiation size threshold. With the thrust force of about 1–10 pN, they can efficiently swim with the mean speed in the range of 50–200 μm s^−1^ at low Reynolds flow regimes.^76^ Such a high thrust force would eventually compete with the acoustic radiation forces and allow them to escape the ultrasonic trapping. Here, we aim to investigate such interactions of active microswimmers with acoustic forces in the wall trapping process. Fig. 6a shows the time-lapse image sequence of microalgae from their natural swimming to the ultrasonically trapped stage. During the first 0.2 seconds, the microalgae were swimming freely in the 200 μm rectangular channel. Then, immediately after the application of ultrasound, they were pushed towards the walls within 1.1 seconds and kept trapped at the boundaries for a longer period without considerable escaping events (Movie S3†). The frequency was tuned to be around 164 kHz with a driving voltage of 64 Vpp. To keep the microalgae trapped stably, the acoustic driving voltage should be tailored to overcome the microswimmers’ thrust force. Fig. 6b shows the simultaneous escaping and trapping events for microswimmers under the driving voltage of 40 Vpp and 64 Vpp, indicated by red and green arrows, respectively (Movie S4†). As expected for higher driving voltage microalgae were all stably trapped at the wall boundaries.

In addition to continuous ultrasonic trapping, a precise wall trapping mode could be possible after fine-tuning the necessary voltage amplitude. Using amplitude modulation, we tested the microalgae behaviour by giving a pulsed actuation of 2 seconds period as shown in Fig. 6c and Movie S5.† The center position of the microalgae in the *x*-axis (along the width of the microchannel) *versus* time is depicted in Fig. 6d. The microalgae trajectory validates the motion control under off/on ultrasound field, although they tend to lag the pulsating signal due to the possible drag force. Furthermore, we investigated the behaviour of the cells while trapped at the boundaries. Due to the presence of the secondary-order streaming forces near the soft walls at large driving voltage of 80 Vpp, the cells rotate, as shown in Fig. 6e and Movie S6.† This phenomenon is similar to the rotational behaviour of cell/particles close to the oscillating microbubbles.^48^ These results suggest the possibility of acoustofluidic wall-trapping of microalgae for in-line bioprocess monitoring and sensing applications. Furthermore, ultrasound field can be used to accelerate the monitoring measurement by aligning cells and prevent bioaccumulation-based contamination of the detection sensors in fermentation cultures.^4^

## Conclusion

We have demonstrated the acoustic manipulation and wall trapping of microparticles and motile cells using flexural-wave based actuation. Unlike the existing wall trapping acoustofluidic devices such as the modified BAW-based design^17^ and SAW-based membrane trapping^39^ method, the proposed approach (1) uses a simple fabrication technique where a standard microscope glass slide is utilized as the substrate for flexural waves propagation; (2) exploits both lateral sidewalls for trapping particles or cells, yielding a higher throughput; (3) does not require precise positioning of the microchannel due to relatively large acoustic wavelength/channel-width ratio which can range between 10 and 150; (4) enables, in principle, the lateral wall trapping inside microchannels of arbitrary cross-section geometry. We showed that, using acoustic wavelength larger than the microchannel width, it is possible to generate sufficient radiation force for manipulating micron-size objects in both rectangular and circular channels with different sizes. The system-level numerical simulations revealed the importance of transverse displacement profile of the glass substrate at resonance and its coupled dynamics with the acoustic radiation and streaming forces inside the microchannel. The particle tracing simulations also confirmed the dominance of the acoustic radiation force over the streaming force for approximately larger than 10 μm size particles. Finally, we investigated the interaction of active microswimmers such as microalgae under ultrasonic trapping forces. We showed that the driving voltage of the acoustic field can be tuned to overcome the thrust force of the microswimmers, which is in the range of 1–10 pN, and trap them at the soft walls. Moreover, a controlled trap-release sequence was shown to be feasible using the amplitude modulated acoustic signal. One of the immediate applications of the soft attractor walls is the rapid surface coating of the cells or particles. For instance, the attraction towards an antibody-coated PDMS wall could enhance the surface functionalization of microalgae microswimmers in a short time period, before deploying them as biohybrid units. The rotation of the cells near the soft walls could also increase the surface-coating efficiency.

## Materials and methods

### Flexural-wave chips

The PDMS microchannels (10 : 1 base monomer to crosslinker ratio) were made using the standard soft lithography process. The rectangular channels with different width of 100–750 μm and height of 100 μm were prepared using the casting, where the negative master mold was made out of SU-8 photoresist. The circular cross-sectional channels were made by embedding wires as the negative master mold with different diameters of 200–500 μm in the PDMS. The admittance-phase measurements were performed using ENA network analyzer (E5061B, Keysight Technologies).

### Cell preparation

*Chlamydomonas reinhardtii* (strain CC-125 wild type mt+), obtained from “chlamycollection.org,“ were used for the experiments as the unicellular green microalgae. The microalgae were cultured in tris-acetate–phosphate (TAP) medium (Thermo Fisher Scientific) under a 12 : 12 h light–dark cycle (Philips MASTER TL-D 58 W/840 Super 80 Weiss) with orbital shaking at 150 rpm at room temperature. The cells were grown to optical density (OD) of 0.3 at 680 nm.

### Imaging setup

The ultrasonic trapping process of the microalgae were monitored in real time using a fluorescence microscope (Eclipse, Ti-E) inverted motorized microscope (Nikon Corp., Tokyo, Japan) in the PDMS channels. The image sequence diagrams were made by stacking frames using a custommade python script.

## Supplementary Material

Supplementary information † Electronic supplementary information (ESI) available. See DOI:10.1039/d0lc00865f

Supplementary movie

Supplementary movie

Supplementary movie

Supplementary movie

Supplementary movie

Supplementary movie

## Figures and Tables

**Table 1:** Material parameters at *T* = 25 °C

Dimension	Symbol	Value
Glass		
Density	*ρ*_G_	2230 kg m^−3^
Longitudinal speed of sound	$c_{{}_{\text{lo}}}^{G}$	5594 m s^−1^
Transverse speed of sound	$c_{\text{tr}}^{G}$	3425 m s^−1^
Poly-dimethylsiloxane (PDMS, 10 : 1)		
Density	*ρ*_PDMS_	920 kg m^−3^
Longitudinal speed of sound	$\rho_{\text{lo}}^{\text{PDMS}}$	1000 m s^−1^
Transverse speed of sound	$c_{\text{tr}}^{\text{PDMS}}$	65 m s^−1^
Elastic modulus^56^	*C*_11_	1.035–*i*0.0026 GPa
Elastic modulus^56^	*C*_12_	1.027–*i*0.0012 GPa
Elastic modulus^56^	*C*_44_	4.31–*i*0.68 MPa
Water		
Density	*ρ*_f_	1000 kg m^−3^
Speed of sound	*c*_f_	1500 m s^−1^
Compressibility^a^	*κ*_f_	444 TPa^−1^
Viscosity	*η*	0.893 mPa s
Thermal conductivity	*k*_th_	0.603 W m^−1^ K^−1^
Specific heat capacity	*C*_p_	4183 J kg^−1^ K^−1^
Specific heat capacity ratio^b^	*γ*	1.014
Thermal diffusivity^c^	*D*_th_	1.43 × 10^−7^ m^2^ s^−1^
Thermal expansion coefficient	*α*	2.97 × 10^−4^ K^−1^
Polystyrene		
Density^57^	*ρ*_p_	1050 kg m^−3^
Speed of sound^58^ (at 20 °C)	*c*_p_	2350 m s^−1^
Poisson’s ratio^59^	*σ*_p_	0.35
Compressibility^d^	*κ*_p_	249 TPa^−1^

a Calculated as $\kappa_{\text{f}}=\frac{1}{\rho_{\text{f}}c_{\text{f}}^{2}}$.

b Calculated as $\gamma =\frac{T_{\text{f}}\alpha^{2}}{\rho_{\text{f}}C_{\text{p}}\kappa_{\text{f}}}+1$.

c Calculated as $D_{\text{th}}=\frac{k_{\text{th}}}{\rho_{\text{f}}C_{\text{p}}}$.

d Calculated as $\kappa_{\text{p}}=\frac{3\left(1-\sigma_{\text{p}}\right)}{1+\sigma_{\text{p}}}\frac{1}{\rho_{\text{p}}c_{\text{p}}^{2}}$ from ref. 60.

**Table 2:** Design parameters of the PDMS channels

Dimension	Symbol	Value
PDMS width	*W*_PDMS_	6 mm
PDMS height	*H*_PDMS_	4 mm
Rectangular channel Widths	*W*_ch_	100, 200, 500, 750 μm
Rectangular channel Height	*H*_ch_	100 μm
Circular channel Diameters	*D*_ch_	200, 300, 400, 500 μm
Circular channel		
Distance from the bottom	*D*_bottom_	100 μm
PDMS and channel length	*L*	8.76 mm

## References

[1] Friend J, Yeo LY (2011). Rev Mod Phys.

[2] Ding X, Lin S-CS, Kiraly B, Yue H, Li S, Chiang I-K, Shi J, Benkovic SJ, Huang TJ (2012). Proc Natl Acad Sci U S A.

[3] Lenshof A, Magnusson C, Laurell T (2012). Lab Chip.

[4] Wiklund M, Radel S, Hawkes JJ (2013). Lab Chip.

[5] Nge PN, Rogers CI, Woolley AT (2013). Chem Rev.

[6] Li P, Mao Z, Peng Z, Zhou L, Chen Y, Huang P-H, Truica CI, Drabick JJ, El-Deiry WS, Dao M (2015). Proc Natl Acad Sci U S A.

[7] Chen M, Cai F, Wang C, Wang Z, Meng L, Li F, Zhang P, Liu X, Zheng H (2017). Adv Sci.

[8] Gu Y, Chen C, Wang Z, Huang P-H, Fu H, Wang L, Wu M, Chen Y, Gao T, Gong J (2019). Lab Chip.

[9] Antfolk M, Kim SH, Koizumi S, Fujii T, Laurell T (2017). Sci Rep.

[10] Park J-W, Lee SJ, Ren S, Lee S, Kim S, Laurell T (2016). Sci Rep.

[11] Antfolk M, Muller PB, Augustsson P, Bruus H, Laurell T (2014). Lab Chip.

[12] Jakobsson O, Grenvall C, Nordin M, Evander M, Laurell T (2014). Lab Chip.

[13] Li S, Ding X, Guo F, Chen Y, Lapsley MI, Lin S-CS, Wang L, McCoy JP, Cameron CE, Huang TJ (2013). Anal Chem.

[14] Guo F, Li P, French JB, Mao Z, Zhao H, Li S, Nama N, Fick JR, Benkovic SJ, Huang TJ (2015). Proc Natl Acad Sci U S A.

[15] Shi J, Ahmed D, Mao X, Lin S-CS, Lawit A, Huang TJ (2009). Lab Chip.

[16] Glynne-Jones P, Boltryk RJ, Hill M, Harris NR, Baclet P (2009). J Acoust Soc Am.

[17] Leibacher I, Schatzer S, Dual J (2014). Lab Chip.

[18] Bachman H, Gu Y, Rufo J, Yang S, Tian Z, Huang P-H, Yu L, Huang TJ (2020). Lab Chip.

[19] Yeo LY, Friend JR (2009). Biomicrofluidics.

[20] Shi J, Yazdi S, Lin S-CS, Ding X, Chiang I-K, Sharp K, Huang TJ (2011). Lab Chip.

[21] Nama N, Barnkob R, Mao Z, Kähler CJ, Costanzo F, Huang TJ (2015). Lab Chip.

[22] Skov N, Bruus H (2016). Micromachines.

[23] Mao Z, Xie Y, Guo F, Ren L, Huang P-H, Chen Y, Rufo J, Costanzo F, Huang TJ (2016). Lab Chip.

[24] Barnkob R, Nama N, Ren L, Huang TJ, Costanzo F, Kähler CJ (2018). Phys Rev Appl.

[25] Skov NR, Sehgal P, Kirby BJ, Bruus H (2019). Phys Rev Appl.

[26] Destgeer G, Sung HJ (2015). Lab Chip.

[27] Shi J, Mao X, Ahmed D, Colletti A, Huang TJ (2008). Lab Chip.

[28] Shilton RJ, Travagliati M, Beltram F, Cecchini M (2014). Adv Mater.

[29] Reichert P, Deshmukh D, Lebovitz L, Dual J (2018). Lab Chip.

[30] Garofalo F, Laurell T, Bruus H (2017). Phys Rev Appl.

[31] Ley MW, Bruus H (2017). Phys Rev Appl.

[32] Hahn P, Lamprecht A, Dual J (2016). Lab Chip.

[33] Leibacher I, Reichert P, Dual J (2015). Lab Chip.

[34] Hahn P, Schwab O, Dual J (2014). Lab Chip.

[35] Muller PB, Barnkob R, Jensen MJH, Bruus H (2012). Lab Chip.

[36] Hill M, Townsend RJ, Harris NR (2008). Ultrasonics.

[37] Glynne-Jones P, Boltryk RJ, Hill M (2012). Lab Chip.

[38] Ding X, Li PS, Lin CS, Stratton ZS, Nama N, Guo F, Slotcavage D, Mao X, Shi J, Costanzo F (2013). Lab Chip.

[39] Collins DJ, Alan T, Neild A (2014). Appl Phys Lett.

[40] Collins DJ, O’Rorke R, Devendran C, Ma Z, Han J, Neild A, Ai Y (2018). Phys Rev Lett.

[41] Devendran C, Choi K, Han J, Ai Y, Neild A, Collins DJ (2020). Lab Chip.

[42] Ahmed D, Baasch T, Blondel N, Läubli N, Dual J, Nelson BJ (2017). Nat Commun.

[43] Haake A, Dual J (2004). Ultrasonics.

[44] Haake A, Dual J (2005). J Acoust Soc Am.

[45] Yeo L, Rezk A (2017). Inf MIDEM.

[46] Ahmed D, Baasch T, Jang B, Pane S, Dual J, Nelson BJ (2016). Nano Lett.

[47] Kaynak M, Ozcelik A, Nama N, Nourhani A, Lammert PE, Crespi VH, Huang TJ (2016). Lab Chip.

[48] Ahmed D, Ozcelik A, Bojanala N, Nama N, Upadhyay A, Chen Y, Hanna-Rose W, Huang TJ (2016). Nat Commun.

[49] Kaynak M, Ozcelik A, Nourhani A, Lammert PE, Crespi VH, Huang TJ (2017). Lab Chip.

[50] Aghakhani A, Yasa O, Wrede P, Sitti M (2020). Proc Natl Acad Sci U S A.

[51] Bruus H (2012). Lab Chip.

[52] Bruus H (2012). Lab Chip.

[53] Auld BA (1973). Acoustic fields and waves in solids.

[54] Leissa AW (1973). J Sound Vib.

[55] Ventsel E, Krauthammer T, Carrera E (2002). Appl Mech Rev.

[56] Moiseyenko RP, Bruus H (2019). Phys Rev Appl.

[57] Haynes WM (2014). CRC handbook of chemistry and physics.

[58] Bergmann L (1954). The ultrasound and its application in science and technology.

[59] Mott P, Dorgan J, Roland C (2008). J Sound Vib.

[60] Landau L (1986). Course of theoretical physics.

[61] Tribler PM (2015). Acoustic streaming in microchannels: The trinity of analytics, numerics and experiments.

[62] Wiklund M, Green R, Ohlin M (2012). Lab Chip.

[63] Koklu M, Sabuncu AC, Beskok A (2010). J Colloid Interface Sci.

[64] Barnkob R (2012). Physics of Microparticle Acoustophoresis: Bridging Theory and Experiment.

[65] Bruus H (2012). Lab Chip.

[66] Rayleigh L (1884). Philos Trans R Soc London.

[67] Gralinski I, Alan T, Neild A (2012). J Acoust Soc Am.

[68] Gralinski I, Raymond S, Alan T, Neild A (2014). J Appl Phys.

[69] Lei J, Cheng F, Li K, Guo Z (2020). Appl Phys Lett.

[70] Alapan Y, Yasa O, Yigit B, Yasa IC, Erkoc P, Sitti M (2019). Annu Rev Control Robot Auton Syst.

[71] Erkoc P, Yasa IC, Ceylan H, Yasa O, Alapan Y, Sitti M (2019). Adv Ther.

[72] Xie S, Wang X, Jiao N, Tung S, Liu L (2017). Lab Chip.

[73] Ye Z, Sitti M (2014). Lab Chip.

[74] Bosma R, van Spronsen WA, Tramper J, Wijffels RH (2003). J Appl Phycol.

[75] Sivaramakrishnan R, Incharoensakdi A (2018). Bioresour Technol.

[76] Yasa O, Erkoc P, Alapan Y, Sitti M (2018). Adv Mater.

