# Supplementary material for: Flexural wave-based soft attractor walls for trapping microparticles and cells†
Source: Lab Chip. Author manuscript; Available in PMC 2022 Apr 26. (PMC7612665; doi:10.1039/d0lc00865f)
Supplement: Supplementary information [file EMS144355-supplement-Supplementary_information.pdf]

Electronic Supplementary Material (ESI) for Lab on a Chip.
This journal is © The Royal Society of Chemistry 2020

Electronic Supplementary Information

Flexural wave-based soft attractor walls for trapping microparticles and cells

Amirreza Aghakhani,[a‡] Hakan Cetin,[ab‡] Pelin Erkoc,[ac], Guney Isik Tombak,[ad] and Metin Sitti[*aef]

[a] *Physical Intelligence Department, Max Planck Institute for Intelligent Systems, 70569 Stuttgart, Germany.*
[b] *Electrical and Electronics Engineering Department, Özyeğin University, 34794 Istanbul, Turkey*
[c] *Faculty of Engineering and Natural Sciences, Bahcesehir University, 34353 Istanbul, Turkey*
[d] *Electrical and Electronics Engineering Department, Boğaziçi University, 34342 Istanbul, Turkey*
[e] *Institute for Biomedical Engineering, ETH Zurich, 8092 Zurich, Switzerland*
[f] *School of Medicine and School of Engineering, Koç University, 34450 Istanbul, Turkey*
‡ These authors contributed equally to this work.
∗ Corresponding author: sitti@is.mpg.de

Supplementary Note 1
Figures S1 to S13
Movies S1 to S6

Supplementary Note 1

In Figure S1, we characterized the locations of PDMS channel and PZT/brass plate on a glass plate of 75 mm length. The diameter of PZT/brass plate is 20 mm, which is almost proportional with the wavelength of higher flexural modes of the glass plate ($\lambda = 2L/n$). We placed the PZT/brass plate a length of its radius away from the end of glass plate. Further, the distance between PZT/brass plate and the center of PDMS channel is determined as the length of PZT/brass plate diameter. This configuration transfers the most of the PZT energy to the channel through the glass plate, where the PZT/brass plate and the PDMS microchannel are aligned by the distance proportional with the wavelength of higher flexural modes of the glass plate.

In Figure S3, we performed the modal analyses of a glass plate and the whole system by using the 2D and 3D modelling. The various flexural modes of a glass plate are examined and we found that the resonance frequencies (f_{M1}, f_{M2}, f_{M3}) are almost the same for the 2D and 3D models as shown in Figure S3a and S3c. In Figure S3b and S3d, it is also shown that the resonance frequencies of the whole system are approximately equal for both 2D and 3D model. In numerical calculations, we used the simplified 2D modelling, where the complexity of the 3D modelling is high.

In Figure S4, we implemented the simplified 2D modelling by defining the flexural waves as the displacement condition on a glass substrate instead of analyzing the whole 2D system shown in Figure S3d. In the 2D numerical model, The displacement condition is defined as the sinusoidal function ($A_1 cos(k_1 x), A_2 sin(k_2 x)$) for two cases, where the wavenumbers ($k = \omega/c$) are calcualated for the corresponding flexural mode from the dispersion curves shown in Figure S10. As the wavelength of flexural wave at low frequency is considerably larger than the width of microchannel, the pressure nodes can be located at the outside of the microchannel. In the first case shown in Figure S4a, the pressure nodes are placed at the PDMS cover by cos displacement profile, where the pressure antinode is alinged at the center of the microchannel. This configuration leads the wall trapping. For the second case shown in Figure S4b, the pressure node is placed at the the center of the microchannel by sin displacement profile. As expected, the particles move towards the center of channel in the second configuration.

Figure S10 shows the wave velocity as a function of frequency in a 1 mm thick glass plate, where we calculated the dispersion curves for the Lamb waves propagating in a plate by using the GUIGUW (Graphical User Interface for Guided Ultrasonic Waves) software[1]. Flexural waves are dispersive whereas longitudinal waves are not where f < 1 MHz. For the operating frequencies 50 kHz $< f <$ 200 kHz, the wavelengths of flexural waves are calculated as 6.6 mm $< \lambda <$ 13.8 mm.

It is important to note that the 2D numerical models presented in Figures S11 and S12 are the exactly same models used for the calculation of particle trajectories presented in Figures 3 and 5, respectively. Figures S11a and S12a show the displacement profiles for the top and bottom channel walls of the rectangular microchannel and the top and bottom semicircle channel walls of the circular microchannel, respectively. In both cases, the cosine displacement profile is applied on the bottom PDMS layer with 105 kHz for the rectangular channnel and 87 kHz for the circular channnel. Further, Figures S11b and S12b show the acoustic fields inside the rectangular and circular microchannels. In our numerical analysis, we get the first order acoustic fields ($p_1, \boldsymbol{v_1}$) to calculate the radiation force ($\boldsymbol{F}^{rad}$) and second order acoustic field ($\boldsymbol{v_2}$) to calculate the drag force($\boldsymbol{F}_{drag}$). Further, Figures S11c and S12c show the $\boldsymbol{v_2}$ inside the channels close to the bottom wall, where the boundary streaming rolls are not observed within the calculated boundary layer thickness. In our configuration, we actuate the bottom channel wall vertically from where the streaming is driven. The velocity decays to zero at the wall due to the no-slip boundary condition. In contrast to hard wall configurations[2], we used lossy soft material (PDMS) in our design which minimizes the velocity gradients near the walls by allowing the first-order velocity to have a slip-velocity. This behavior was also observed in the study defined the PDMS channel walls as lossy boundary conditions[3].

References

[1] A. Marzani, P. Bocchini, E. Viola, I. Bartoli, S. Coccia, S. Salamone and F. L. di Scalea, attidella 13a Congresso Nazionale sulle prove non distruttive Monitoraggio e diagnostica AIPnD, Roma, Italia, 2009, pp. 1–9.

[2] P. B. Muller, R. Barnkob, M. J. H. Jensen and H. Bruus, *Lab on a Chip*, 2012, **12**, 4617–4627.

[3] N. Nama, R. Barnkob, Z. Mao, C. J. Kähler, F. Costanzo and T. J. Huang, *Lab on a Chip*, 2015, **15**, 2700–2709.

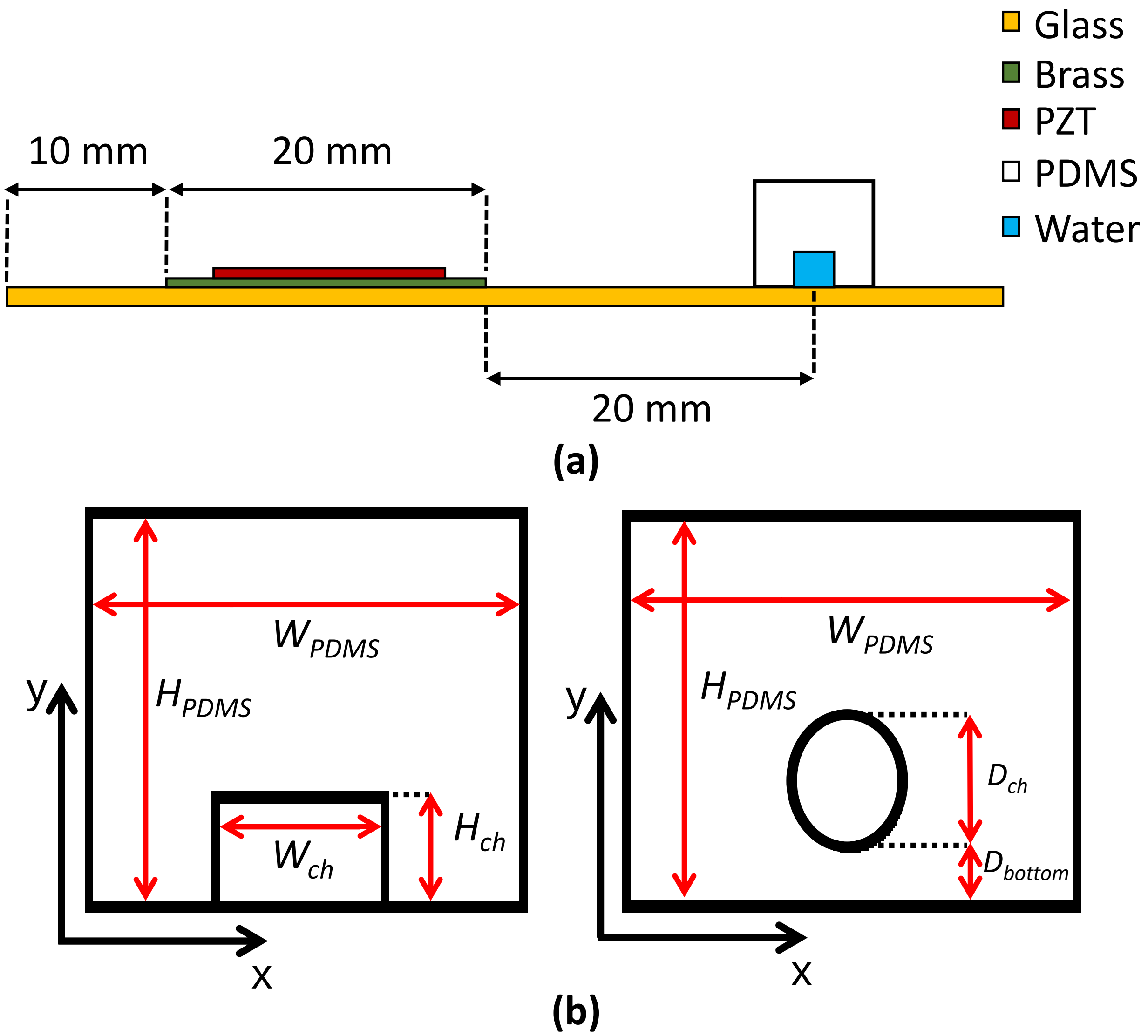

Figure S1 (a) Locations of PDMS channel and PZT on the glass plate and (b) design parameters of rectangular and circular channels.

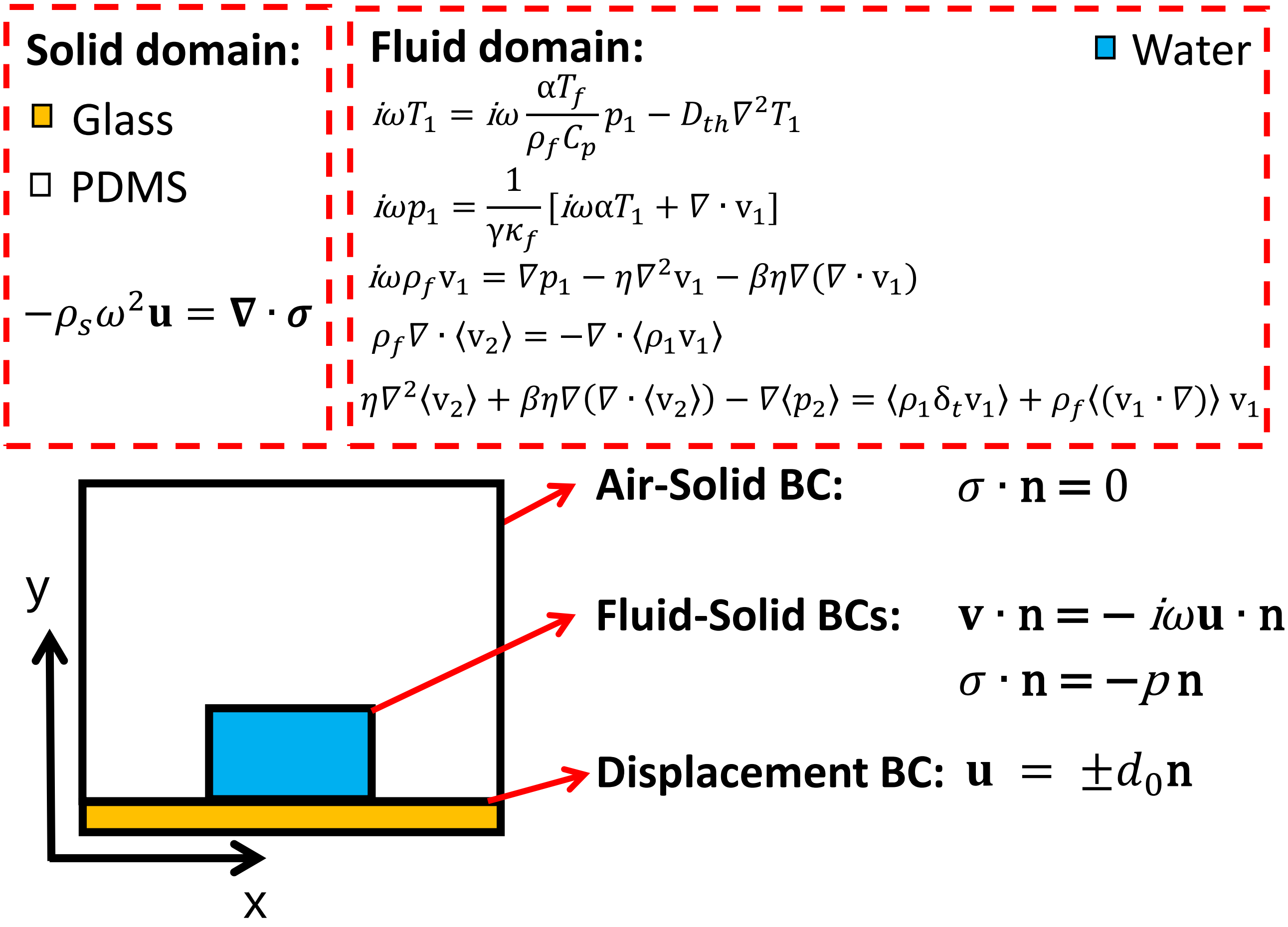

Figure S2 Boundary conditions and computational domains for the PDMS channel.

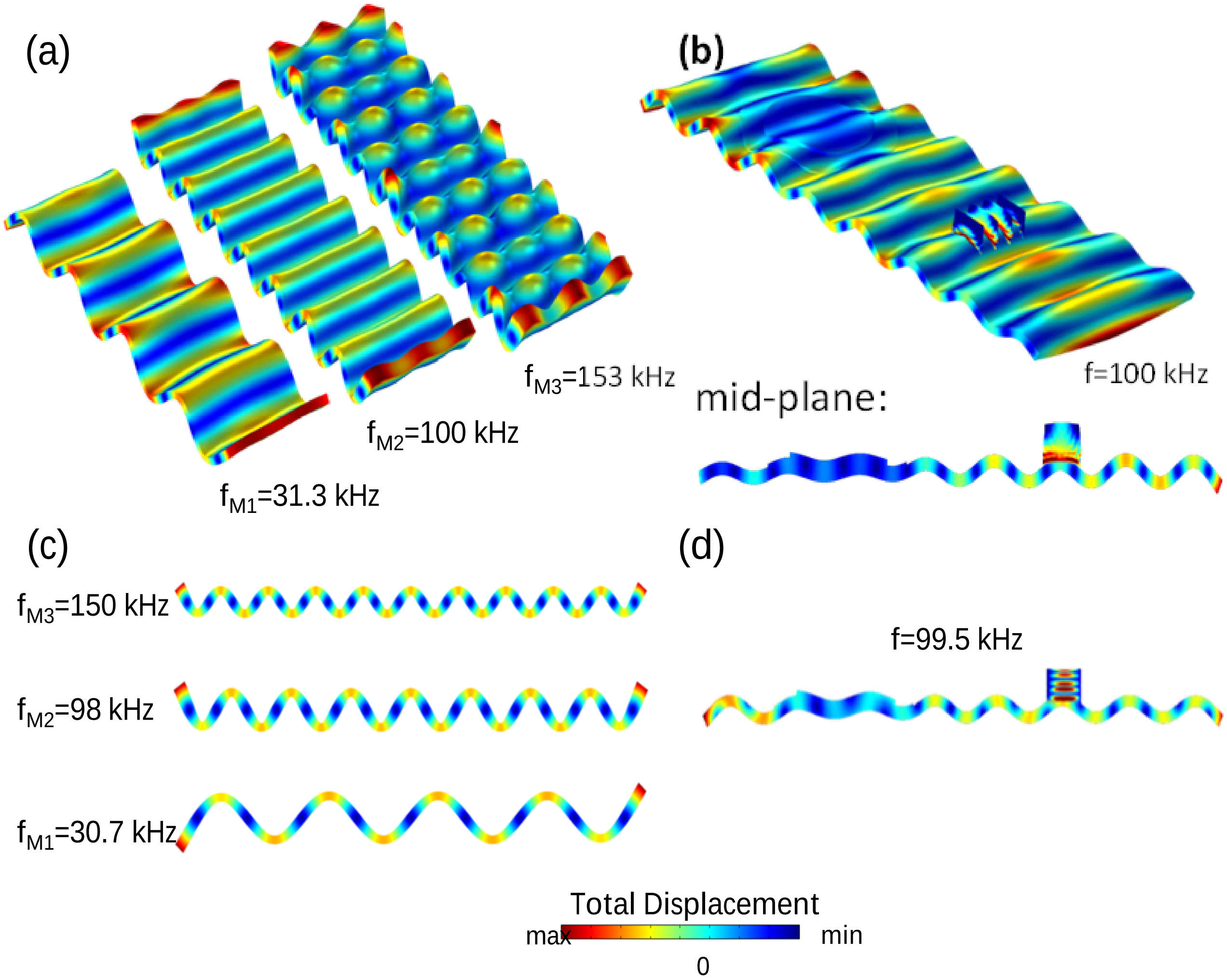

Figure S3 3D Mode shapes of (a) the glass plate, (b) the whole system and its mid-plane cross-section representing 2D mode shapes of (c) the glass plate and (d) the whole system.

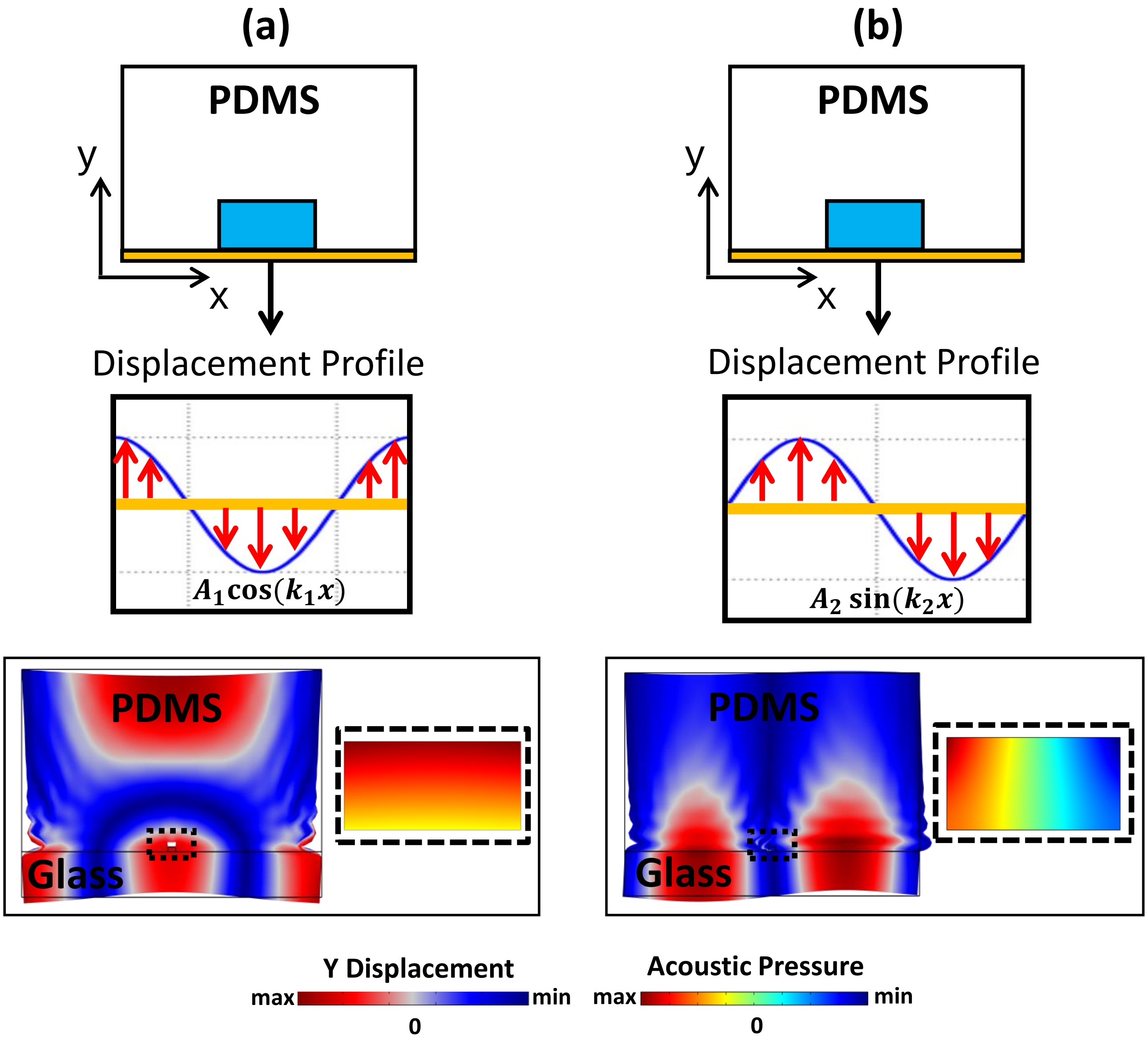

Figure S4 Two types of sinusoidal displacement profiles for the implementation of pressure distribution inside the water channel by aligning (a) the pressure antinode and (b) the pressure node at the center of PDMS channel.The operating frequencies are 105 kHz for the cosine displacement profile and 155 kHz for the sine displacement profile.

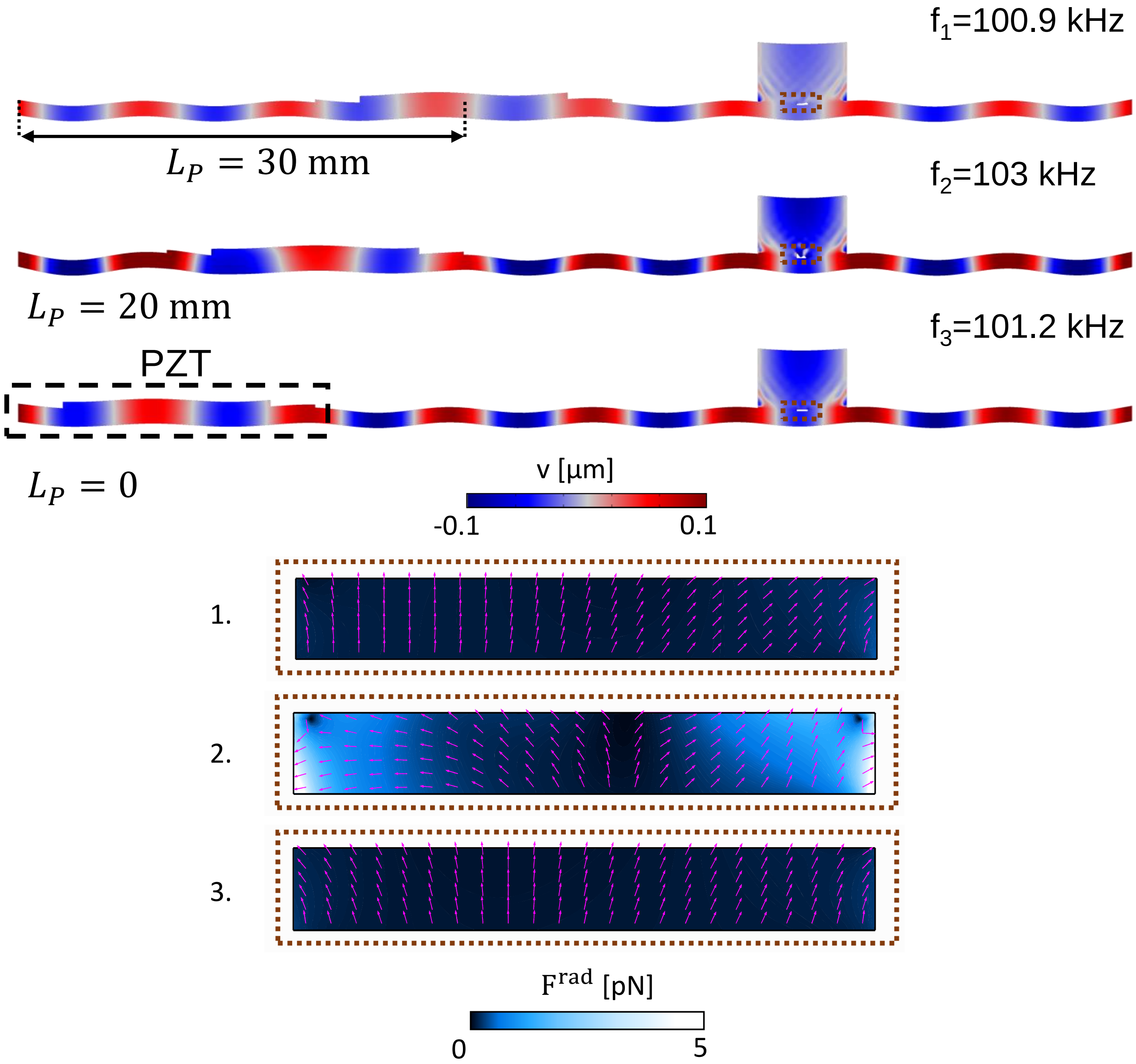

Figure S5 Characterization of the PZT position for the same flexural mode of the whole system, while the position of the PDMS channel on the glass plate is fixed. The resonance frequencies of the system change slightly due to variation in mass and stiffness components of the system by moving the PZT location. The channel width is 750 μm.

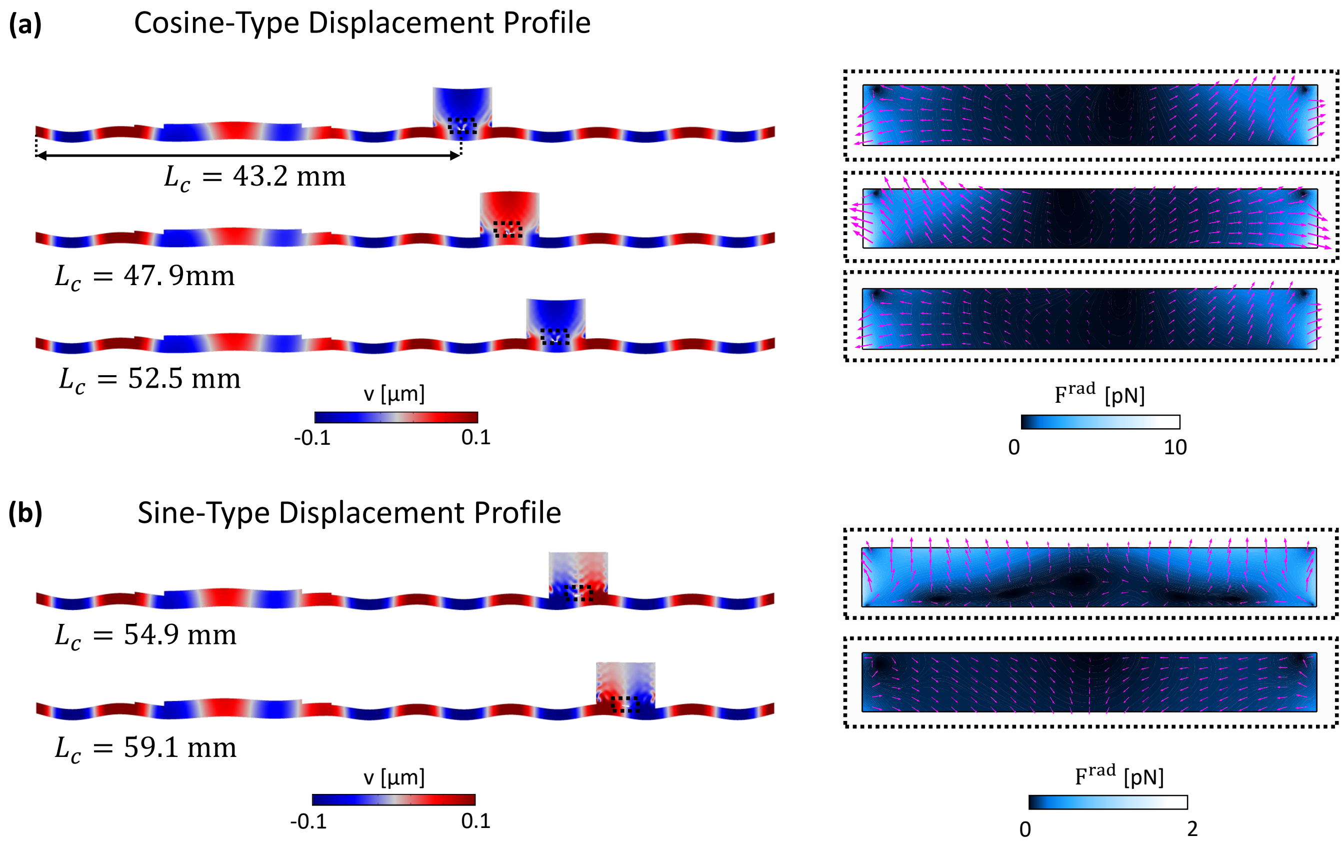

Figure S6 Characterization of the PDMS channel position for (a) cosine-type displacement profile, rendering wall trapping of particles, and (b) sine-type displacement profile, where the operating frequency equals to 103 kHz for the 750 μm width channel. The simulations indicate that the similar acoustic manipulation is possible by moving the center of PDMS location on maximum- or zero-amplitude bending mode positions.

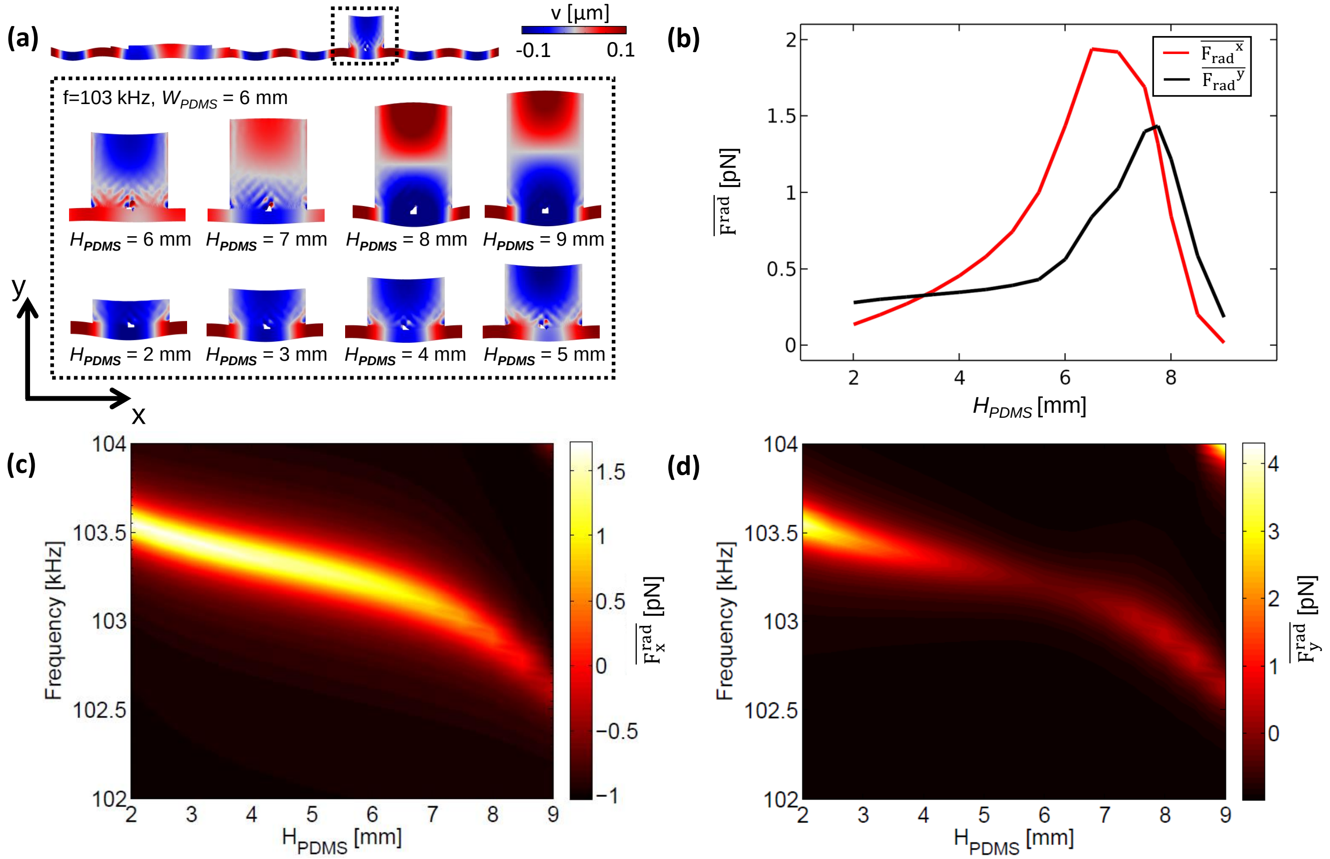

Figure S7 Characterization of the PDMS height (H_{PDMS}), where the operating frequency is 103 kHz and W_{PDMS} = 6 mm. (b) Spatially averaged radiation force ($\overline{\boldsymbol{F}^{rad}}$) inside the 500μm width channel is calculated for 10 μm particles, where H_{PDMS} = 2-9 mm. Heat maps of (c) x-component of spatially averaged radiation force ($\overline{F_x^{rad}}$) and (d) y-component of spatially averaged radiation force ($\overline{F_y^{rad}}$) are calculated for the PDMS heights in the range of 2 to 9 mm and the operating frequency range of 102 to 104 kHz. The maximum radiation force shifts by the change in the height of the PDMS channel due to the slight shift of the resonance frequency of the whole system.

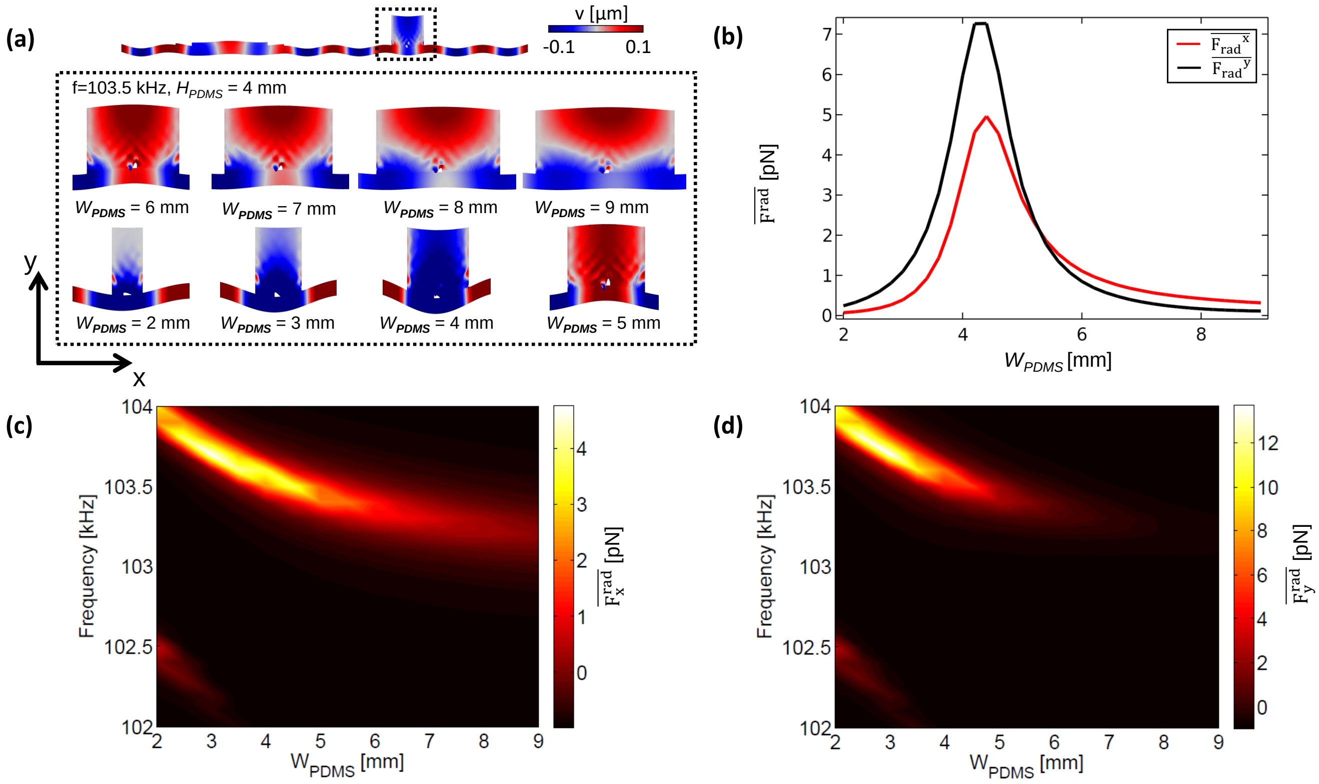

Figure S8 (a) Characterization of the PDMS width (W_{PDMS}), where the operating frequency is 103.5 kHz and H_{PDMS} = 4 mm. (b) Spatially averaged radiation force ($\overline{\boldsymbol{F}^{rad}}$) inside the 500μm width channel is calculated for 10 μm particles, where W_{PDMS} = 2-9 mm. Heat maps of (c) x-component of spatially averaged radiation force ($\overline{F_x^{rad}}$) and (d) y-component of spatially averaged radiation force ($\overline{F_y^{rad}}$) are calculated for the PDMS widths in the range of 2 to 9 mm and the operating frequency range of 102 to 104 kHz.The maximum radiation force shifts by the change in the width of the PDMS channel due to the slight shift of the resonance frequency of the whole system.

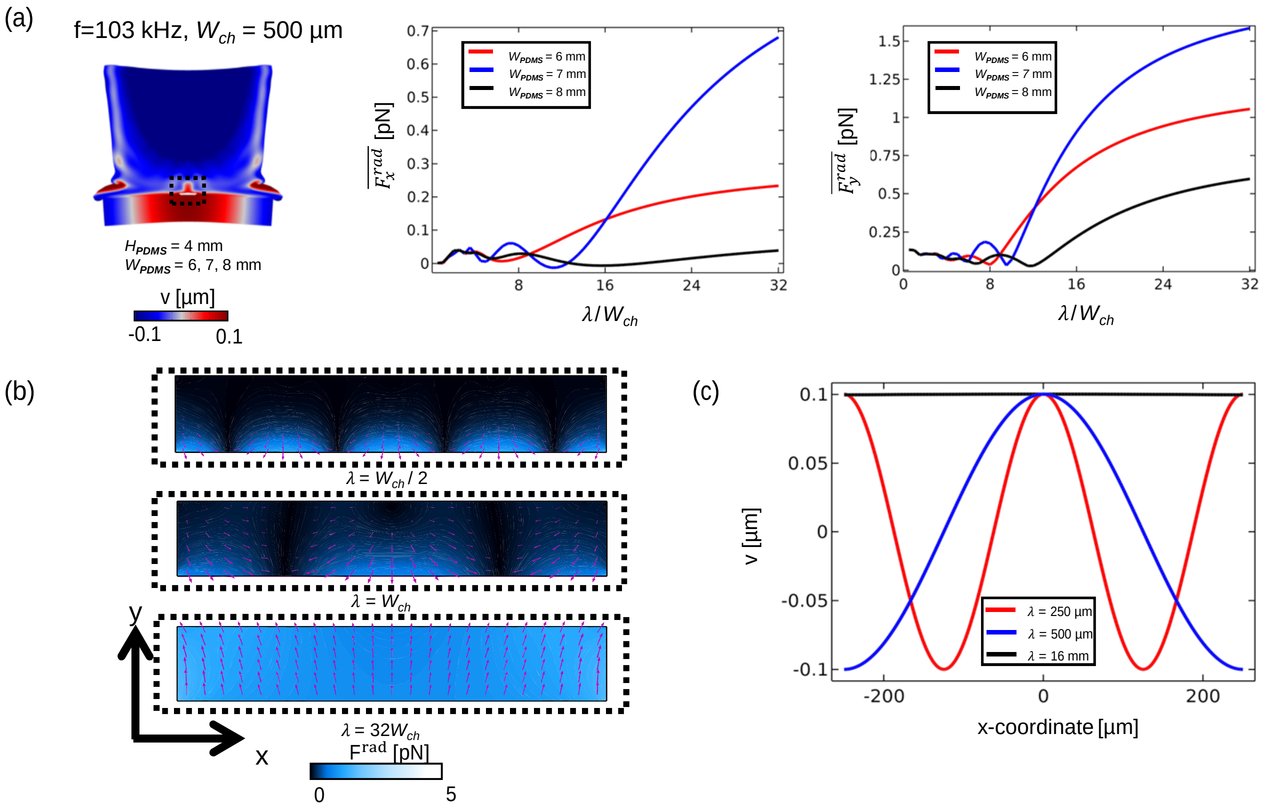

Figure S9 (a) Characterization of the acoustic wavelength (λ), where the displacement of $1 \times 10^{-7} cos(kx)$ is applied on the bottom PDMS layer with the operating frequency of 103 kHz. The spatially averaged radiation force ($\overline{\boldsymbol{F}^{rad}}$) is calculated for 10 µm particles, where λ = 0.25-16 mm, H_{PDMS} = 4 mm and W_{PDMS} = 6, 7, 8 mm. (b) Radiation force field of the 500 µm width channel for several wavelength/width ratios. (c) The y-displacement profiles of bottom channel wall are presented, where λ equals to 250 µm, 500 µm and 16 mm.

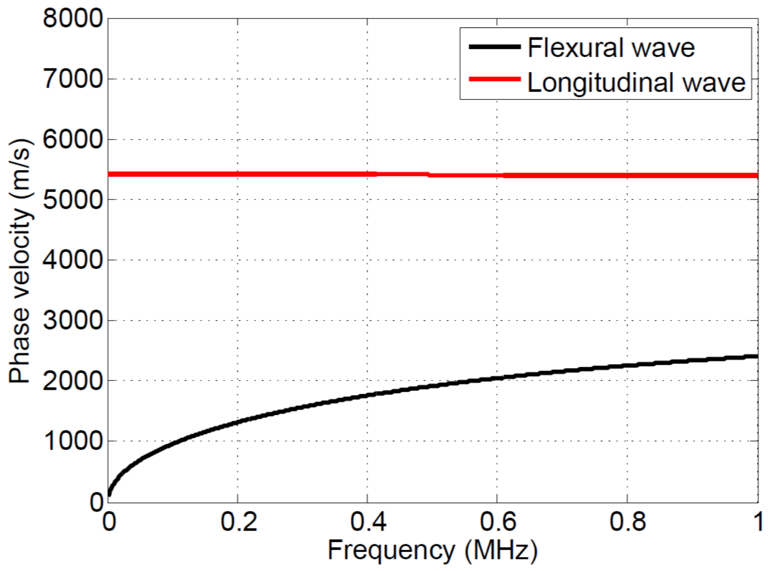

Figure S10 Dispersion curves for a 1 mm thick glass plate.

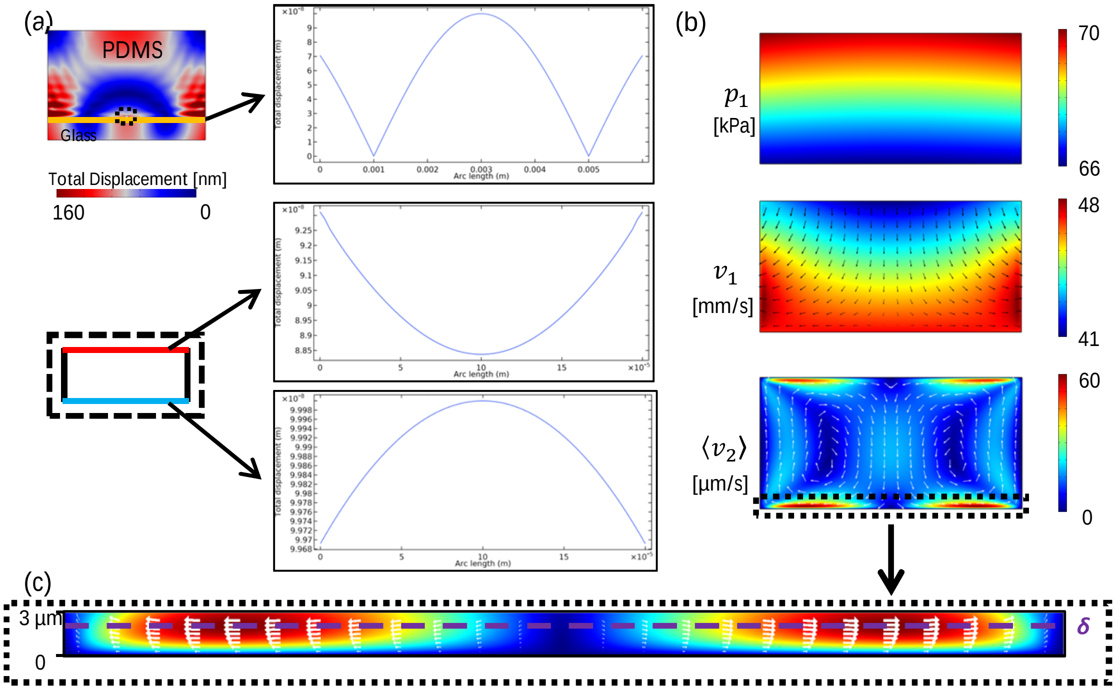

Figure S11 Total displacement profiles of bottom PDMS layer, top and bottom channel wall, and (b) color plots of the first order pressure field p_1 velocity field $\boldsymbol{v_1}$ and the time averaged second order velocity field $< \boldsymbol{v_2} >$, where the displacement of $1 \times 10^{-7} cos(kx)$ is applied on the bottom PDMS layer, and (c) zoom of the time-averaged second-order velocity field $< \boldsymbol{v_2} >$ in (b), where the calculated boundary layer thickness (δ) equals to 1.69 µm. The operating frequnecy is 105 kHz for the circular channel of 200 µm width.

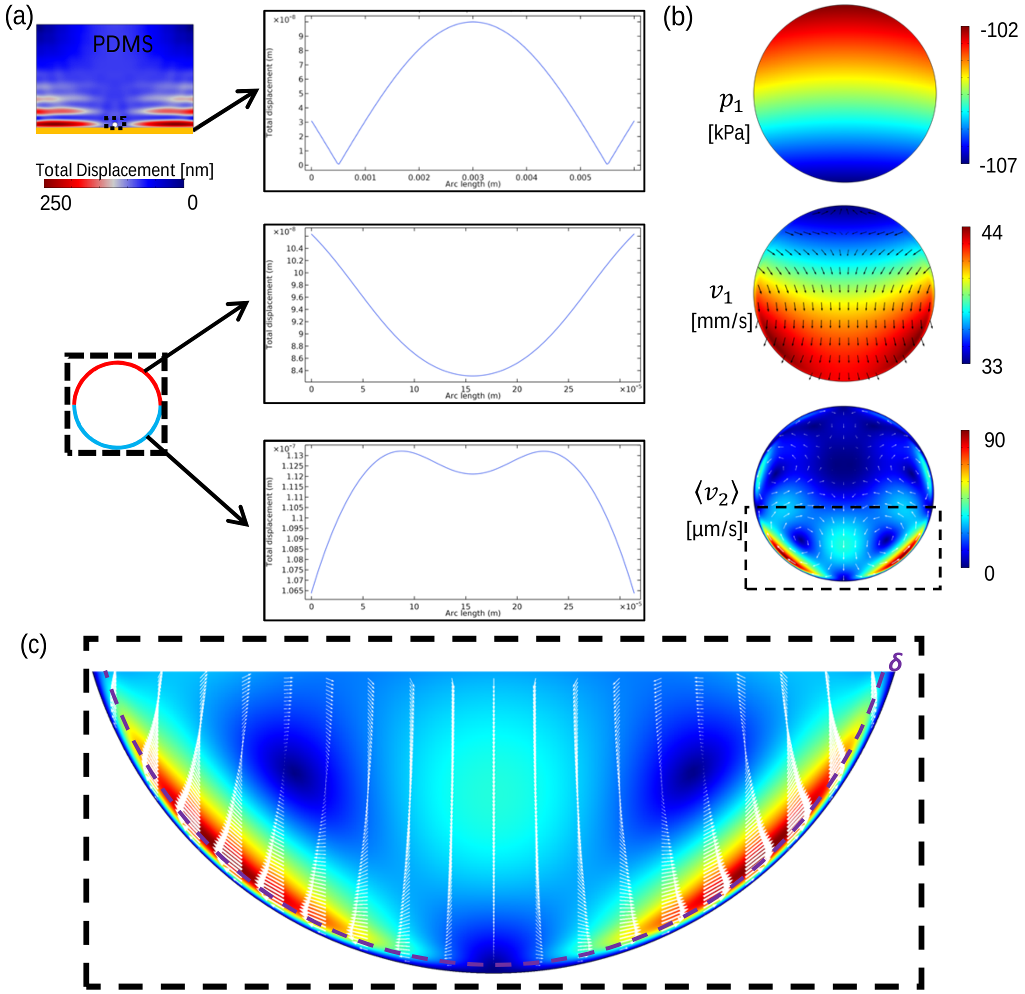

Figure S12 Total displacement profiles of bottom PDMS layer, top and bottom semicircle channel wall, and (b) color plots of the first order pressure field p_1 velocity field $\boldsymbol{v_1}$ and the time averaged second order velocity field $< \boldsymbol{v_2} >$, where the displacement of $1 \times 10^{-7} cos(kx)$ is applied on the bottom PDMS layer, and (c) zoom of the time-averaged second-order velocity field $< \boldsymbol{v_2} >$ in (b), where the calculated boundary layer thickness (δ) equals to 1.69 μm. The operating frequnecy is 87 kHz for the circular channel of 200 μm diameter.

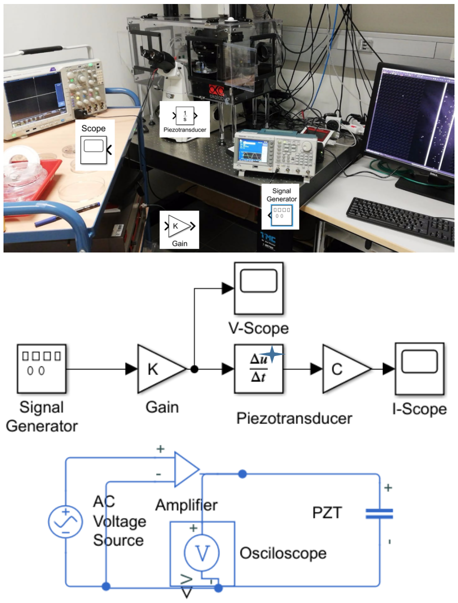

Figure S13 Experimental setup (top) and the open-loop block representation (bottom).
